# Advanced Proteomics Approaches Hold Potential for the Risk Assessment of Metabolism-Disrupting Chemicals as Omics-Based NAM: A Case Study Using the Phthalate Substitute DINCH

**DOI:** 10.1021/acs.est.5c01206

**Published:** 2025-07-30

**Authors:** Alix Sarah Aldehoff, Isabel Karkossa, Helen Broghammer, Sontje Krupka, Juliane Weiner, Cornelius Goerdeler, Rima Nuwayhid, Stefan Langer, Martin Wabitsch, Ulrike Rolle-Kampczyk, Nora Klöting, Matthias Blüher, John T. Heiker, Martin von Bergen, Kristin Schubert

**Affiliations:** † Department of Molecular Toxicology, 28342 Helmholtz-Centre for Environmental Research GmbH (UFZ), Leipzig 04318, Germany; ‡ Helmholtz Institute for Metabolic, Obesity and Vascular Research HI-MAG, Helmholtz-Centre Munich at the University of Leipzig and University Hospital, Leipzig 04103, Germany; § Department of Medicine, Endocrinology and Nephrology, University of Leipzig, Leipzig 04103, Germany; ∥ Department of Orthopaedic, Trauma and Plastic Surgery, Division of Plastic, Aesthetic and Special Hand Surgery, University Hospital Leipzig, Leipzig 04103, Germany; ⊥ Division of Paediatric Endocrinology and Diabetes, University Hospital for Children and Adolescents Ulm, Ulm 89075, Germany; # German Centre for Child and Adolescent Health (DZKJ), Leipzig 04103/04318, Germany; ∇ Institute of Biochemistry, Faculty of Biosciences, Pharmacy and Psychology, University of Leipzig, Leipzig 04103, Germany; ○ German Centre for Integrative Biodiversity Research (iDiv) Halle-Jena-Leipzig, Leipzig 04103, Germany

**Keywords:** DINCH, MINCH, metabolic disruption, adipose tissue, adipocytes, acetylation, phosphorylation, thermal proteome profiling

## Abstract

The concept of metabolic disruption through exposure to chemicals has expanded our understanding of how environmental pollution can contribute to metabolic dysregulation and, ultimately, diseases like obesity. New strategies for assessing the risks posed by chemicals are needed, and omics technologies, including proteomics, have proven to be powerful tools for investigating the molecular mechanisms of these metabolism-disrupting chemicals (MDCs). A potential MDC is the plasticizer DINCH—an alternative to legacy phthalates like DEHP, whose primary metabolite MINCH has been linked to the induction of adipogenesis and lipid accumulation. Here, global proteomics was complemented with insights into protein thermal stability and the profiles of post-translational modification (PTM) acetylation and phosphorylation to provide a profound understanding of chemical-induced metabolic disruption in adipocytes. We demonstrate the utility of advanced proteomics approaches in assessing the effects of potential MDCs by using the human SGBS adipocyte cell line. Adipose tissue PTM data from dietary DINCH-exposed mice were assessed as an *in vivo* model, and *in vitro* data shed light on DINCH’s molecular effects, including protein interactions beyond its primary target PPARγ. The results emphasize the potential of omics approaches to enhance current risk assessment frameworks for emerging contaminants.

## Graphical abstract

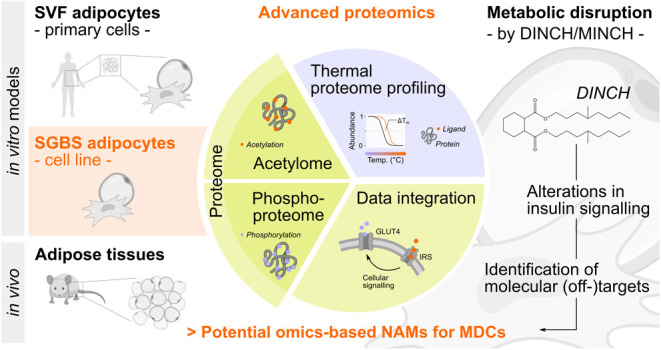

## Introduction

The concept of metabolism-disrupting chemicals (MDCs) has broadened the understanding of chemical-related health impacts on metabolic diseases in recent years.^1,2^ The interference of MDCs with metabolic regulation has been associated with conditions like the metabolic syndrome encompassing abdominal obesity, insulin resistance, hypertension, dyslipidaemia, and hyperglycemia, thereby increasing the risk for type 2 diabetes, cardiovascular, liver, and inflammatory diseases.^3^ In recent decades, the prevalence of metabolic diseases,^4^ including obesity in adults and children,^5^ has increased dramatically in most countries around the world, rendering it a highly concerning global public health burden of pandemic dimensions.^6^ Although obesity ultimately represents an imbalance between the energy supply and demand, the majority of cases cannot be attributed to a single inherent biological cause (monogenic) or lifestyle factor. In contrast, prevalent polygenic forms of obesity with multiple mild mutations that increase susceptibility to metabolic disorders are particularly vulnerable to external influences that can initiate or promote obesity^7^—possibly through exposure to MDCs.^8^

While we are exposed to chronic low doses of a variety of pollutants that are released into the environment by human activities all the time,^9,10^ current risk assessment strategies are not yet equipped with a sufficient regulatory framework to assess potential metabolic disruption of chemicals.^11^ Especially, emerging contaminants are a major threat to societal health as they often replace restricted or regulated chemicals, while maintaining a high functional and/or structural similarity to their predecessor.^12,13^ For instance, diisononyl-cyclohexane-1,2-dicarboxylate (DINCH) with its primary metabolite monoisononyl-cyclohexane-1,2-dicarboxylate (MINCH),^14^ which represents a prominent replacement for legacy phthalates (e.g., di­(2-ethylhexyl) phthalate, DEHP) in many polyvinyl chloride (PVC) consumer products,^15,16^ has been shown to promote adipogenesis and lipid accumulation in human adipocytes using proteomics^17^ and metabolomics.^18^

The analysis of data from omics technologies, such as transcriptomics or metabolomics, displays attractive new approach methods (NAMs) that allow comprehensive molecular insights into chemical-exposed systems, while reducing the burden of existing animal-intensive testing guidelines.^19–22^ Likewise, the OECD has recognized the application of “*omics*” as risk assessment tools,^23,24^ and is undertaking directed efforts to review metabolic disruption by chemicals as a distinct end point under the OECD Test Guidelines Programme (TGP).^25^ The potential of proteomics was only recently focused on evaluating chemical effects and safety, as it allows a close to phenotype insight into the molecular mechanisms triggered upon chemical exposure.^26,27^ This information can be refined by including signatures of post-translational modifications (PTMs) in the proteomic analysis.^28^ The importance of PTMs for adipocyte biology is well-known.^29,30^ They appear highly dynamic during adipocyte development^31^ and provide a versatile system to modify protein structure, activity, localization, or interactions.^29^ Identification of modes of action and underlying molecular initiating events of exposure to chemicals, especially those of emerging concern, will be facilitated by the identification of hub proteins through established bioinformatic analysis (e.g., *weighted gene correlation network analysis* (WGCNA)^32^), thereby enabling a profound understanding of the adverse effects of their everyday presence.

Here, we demonstrate the utility of advanced proteomics approaches, including the phosphoproteome and acetylome, as well as data on protein thermal stability, in a case study for the assessment of chemicals with potentially metabolism-disrupting traits such as DINCH and its primary metabolite MINCH. The suitability of the human Simpson–Golabi–Behmel syndrome (SGBS) adipocyte cell line as a new approach method for targeted metabolomics has been shown recently.^18^ We demonstrate the use of this model for the generation of in-depth proteomics data and provide a comparison to primary human stromal vascular fraction (SVF) adipocytes as well as visceral and subcutaneous adipose tissue proteome data of an orally DINCH-exposed obese mouse model. This data aims to advance our knowledge of the molecular effects of DINCH and its modes of action by the identification of potential chemical–protein interactors beyond the known target PPARγ and demonstrate the applicability of SGBS adipocytes in combination with advanced proteomics approaches as a tool for omics-based NAMs.

## Results

### Adipogenic Effect of DINCH *In Vitro* Is Consistent in Human Subcutaneous Adipose Tissue SVF Cells

To confirm the applicability of the SGBS adipocyte model in terms of similarity to the response of primary adipocytes when studying metabolism-disrupting properties of the primary DINCH metabolite MINCH,^17^ we applied exposure conditions of 10 nM and 10 μM DINCH and MINCH, respectively, to primary stromal vascular fraction (SVF) cells from human subcutaneous adipose tissues (SC AT) that remain to be the gold standard in *in vitro* adipocyte research. The SVF cells were isolated and underwent adipogenic differentiation for 12 days (Figure 1A), similar to the protocol used to differentiate the model SGBS cells. The SVF cells were derived from seven different donors, including male and female subjects, different adipose depot origins (abdominal, leg, and back), and subjects with BMI below and above 30 (Figure 1B). Thus, it should be noted that the sample size is limited to a heterogeneous *n* = 7, for reasons of interpretability and significance. The differentiation control containing rosiglitazone induced adipogenesis and concomitant lipid accumulation (Figure 1C,D). SVF cells exposed to MINCH at 10 nM and 10 μM exhibited a partially differentiated adipocyte phenotype and significantly elevated AdipoRed-stained lipid accumulation when compared to the solvent condition (Figure 1C,D), yet less pronounced than the differentiation control. DINCH exposure did not induce lipid accumulation significantly.

**Figure 1:**
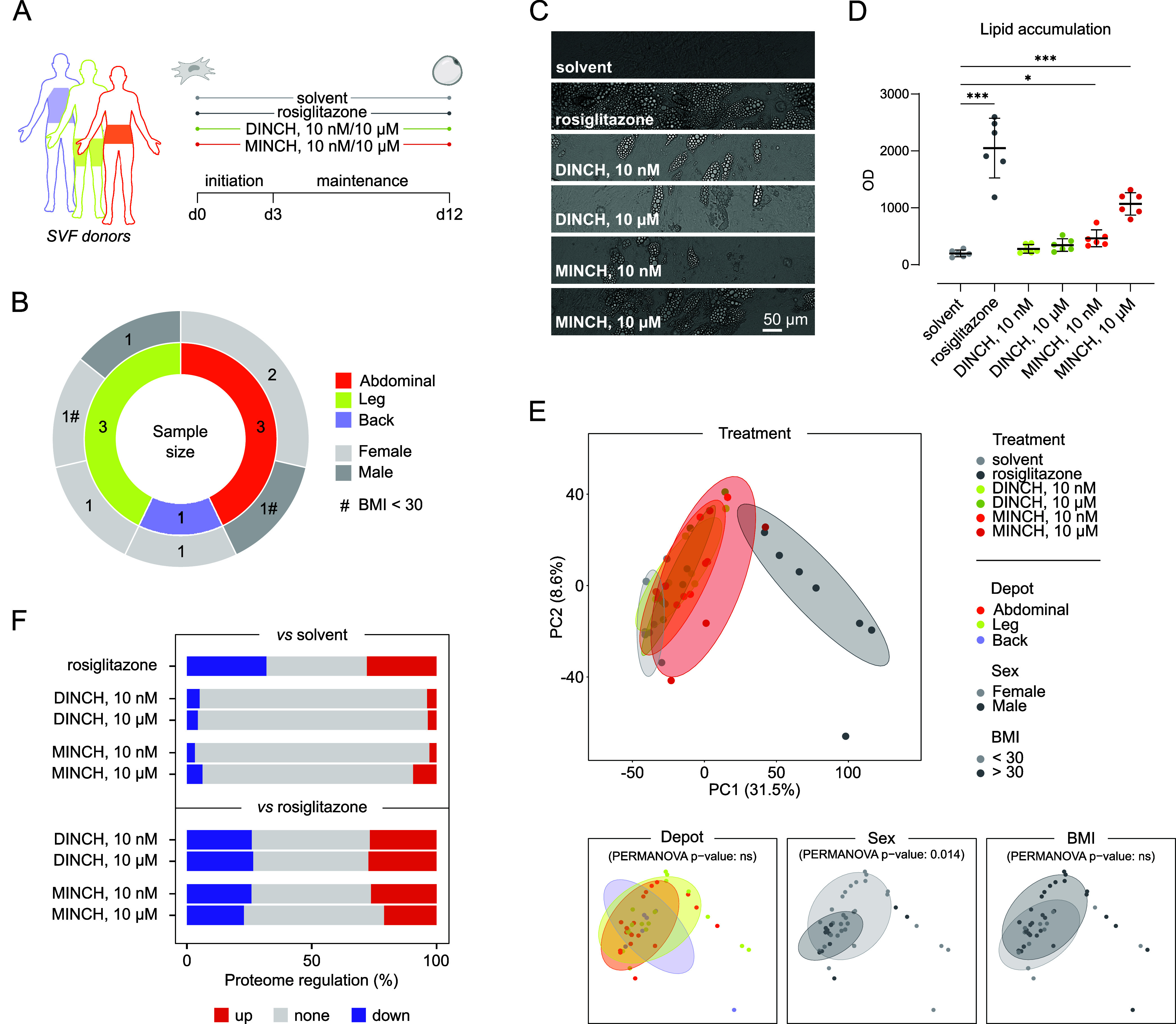
Exposure of DINCH and MINCH to primary human SVF cells from different donors. (A) SVF origins and experimental setup. The SVF cells were derived from different adipose tissue depots of back, leg, or abdominal origin and were subsequently differentiated under different exposure scenarios for 12 days. (B) Overview of sample set and size, *n* = 7. (C) Images of the cells at day 12 of exposure. The scale bar refers to 50 μm. (D) Lipid accumulation under different conditions by AdipoRed staining. Data is displayed as replicates (*n* = 6) with mean ± SD. Significance was calculated using a one-way ANOVA with Brown–Forsythe and Welsh correction. *p* value ≤ 0.05, *; ≤ 0.001, ***. (E) Principal component analysis of proteomic data. Data set separation by treatment, SVF depot origin, donor sex, and BMI range. (F) Proteome regulation of the differentiation exposure scenarios compared to the solvent and differentiation control (rosiglitazone). Proteins quantified in at least 5 out of 7 replicates were considered for analysis. BMI, body mass index; DINCH, diisononyl-cyclohexane-1,2-dicarboxylate; MINCH, monoisononyl-cyclohexane-1,2-dicarboxylate; OD, optical density; PC, principal component; SVF, stromal vascular fraction.

We identified changes in protein abundance after 12 days of SVF adipocyte differentiation as a result of DINCH and MINCH exposure by mass spectrometry-based proteomic analysis. Across all conditions, 5659 proteins were reliably quantified in at least five biological replicates, corresponding to one donor each. Dimensionality reduction by principal component analysis (PCA) indicated a clear separation of the rosiglitazone-exposed from all other samples, despite all donor differences (Figure 1E). Similarly, the DINCH- and MINCH-exposed samples showed separation from the solvent-treated samples, which was most pronounced for the 10 μM MINCH-exposed sample set (Figure 1E). While the SVF depot origin and BMI of the donor did not cause a significant separation of the samples, the respective sex led to a significant separation of the sample set across all exposure conditions (PERMANOVA *p* value 0.014; Figure 1E). Analysis of differential abundance revealed most changes for the differentiation compared with the solvent-treated control (Figure 1F). The relative amount of differentially abundant proteins (DAPs) under the chemically exposed conditions indicated most changes for 10 μM MINCH exposure, while both DINCH and the lower MINCH conditions did not cause a substantial proportion of DAPs when compared to the solvent control (Figure 1F). Conversely, compared to the differentiation control with rosiglitazone, the 10 μM MINCH condition appeared with the lowest proportion of DAPs, indicating an attenuated phenotype of the rosiglitazone treatment.

Overall, 3691 proteins were found with differential abundance (FDR ≤ 0.05) for at least one comparison. A soft clustering approach using the R package *Mfuzz* was applied to assign them to prominent abundance profiles across the experimental conditions, as shown in the stacked clustered heatmaps and fuzz plots (Figure 2A,B). They demonstrate distinct abundance changes with module memberships >0.2 along the differentiation conditions (Figure 2B). The most pronounced features in the abundance changes appear between the solvent and differentiation control (Figure 2B). Cluster 2, being the largest of all, indicates a positive abundance change for the differentiation control and a positive trend in the 10 μM MINCH-exposed samples (Figure 2B). A STRING enrichment using GOBP, GOCC, and KEGG terms, as well as UniProt Keywords, indicates a significant enrichment of cluster 2 members for central metabolic pathways related to mitochondrial energy production, fatty acid, and lipid metabolism, as well as PPAR signaling (Figure 2C). Clusters 7 and 8 appear specific for the DINCH and MINCH treatment conditions. While cluster 7 comprises those proteins whose abundance decreases upon chemical treatment, cluster 8 members showed the opposite increase in abundance (Figure 2B). Significantly enriched terms and keywords for cluster 7 are translation-related, including protein biosynthesis, the proteasome complex, and vesicle trafficking. Cluster 8 indicates significant enrichment for DNA replication and cell division but also endocytosis, insulin secretion, and thyroid hormone signaling (Figure 2C). A complete cluster enrichment overview can be found in the Supporting Information (Figure S1).

**Figure 2:**
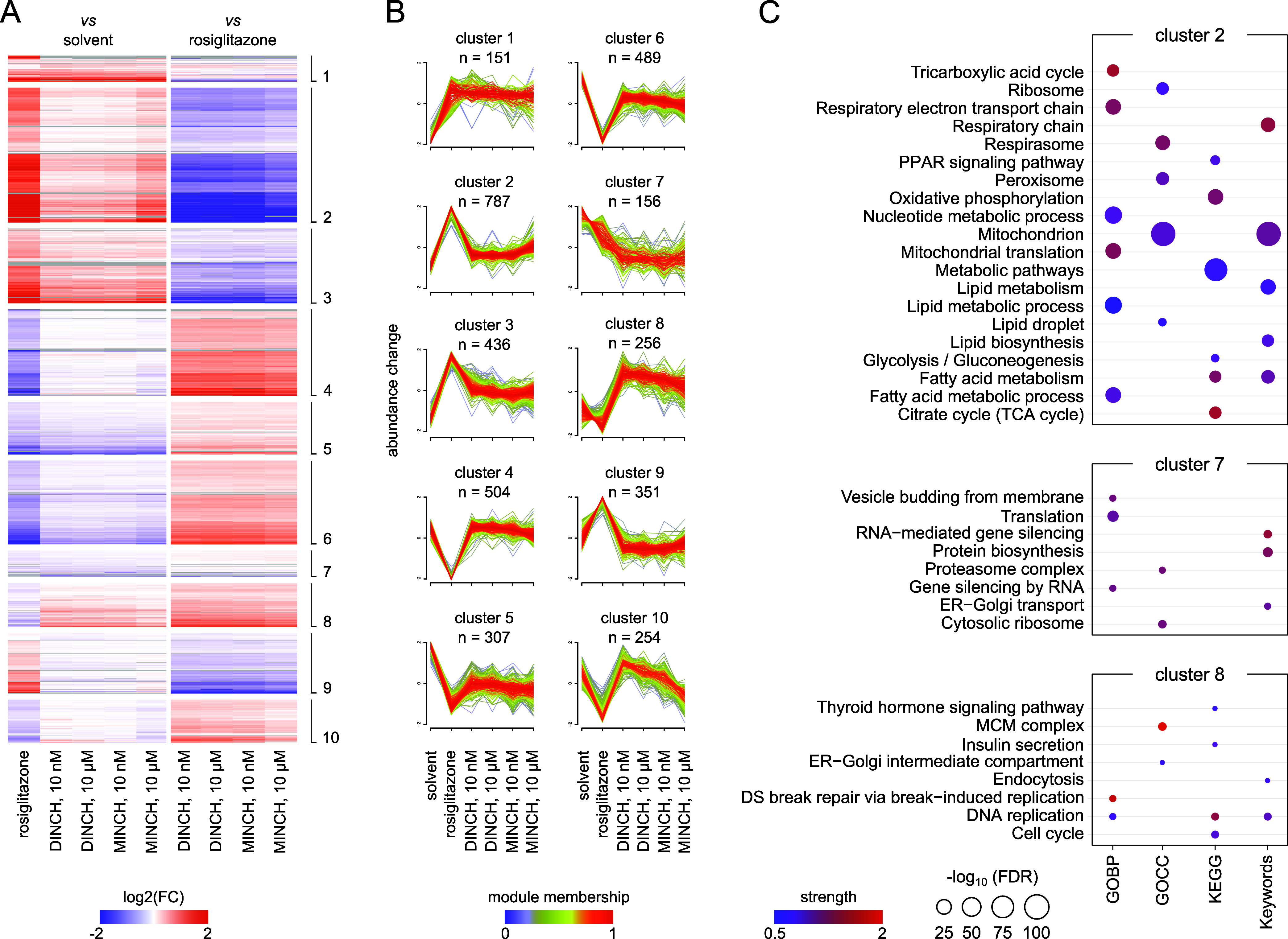
Clustering and enrichment analysis of differentially abundant proteins during DINCH- and MINCH-exposed SVF cell differentiation. (A) Clustered heatmap indicating log_2_ (fold changes) of the comparisons with solvent and differentiation control (rosiglitazone). Displayed are differentially abundant proteins (DAP) only, with FDR ≤ 0.05 for at least one comparison. The different clusters are indicated on the right. (B) Soft clustering of DAP abundance changes across exposure conditions using the R package *Mfuzz*. Abundance profiles with module membership >0.2 are displayed. (C) STRING enrichment of proteins assigned to clusters 2, 7, and 8 using GOBP, GOCC, KEGG terms, and UniProt Keywords. Significantly enriched terms and keywords are displayed as the strength of enrichment and -log_10_(FDR). The complete cluster enrichment is presented in the Supporting Information (Figure S1). FC, fold change; FDR, false discovery rate; GOBP, Gene Ontology Biological Processes; GOCC, Gene Ontology Cellular Components; and KEGG, Kyoto Encyclopedia of Genes and Genomes.

Overall, proteomic data from DINCH- and MINCH-exposed human primary SVF cells show consistent results with those observed in SGBS cells after exposure to the same experimental conditions.^17^ Additionally, the proteomes of both adipocyte models display a good correlation in a Pearson correlation analysis, exemplarily shown for 10 μM MINCH exposure (Figure S2), thereby confirming the relevance of the results obtained in the SGBS cell line for primary adipocytes.

### Identification of Novel Putative Protein Interaction Partners of MINCH in Differentiating SGBS Cells

The results obtained from primary human SVF cells strongly resemble those seen for the SGBS adipocyte cell model, also seen in a good correlation between SVF and SGBS proteomes (Figure S2). To generate a sufficient amount of cell material, the subsequent experiments were performed in SGBS adipocytes. The metabolism-disrupting characteristics of the primary DINCH metabolite MINCH are so far predominantly attributed to an interaction of MINCH with PPARγ.^17^ Yet, a drug or chemical typically interacts with numerous protein targets on a proteome-wide scale, leading to multiple possible modes of action.^33,34^

Here, we performed a *thermal proteome profiling* (TPP) approach to elucidate possible protein interaction partners of MINCH in SGBS adipocytes, reaching beyond the interaction with PPARγ. As adipogenesis progresses, the expression of genes and the abundance of their protein translation products drastically shift.^31^ Thus, studying for protein interactions at one selected time point does not adequately represent all putative interactions with MINCH, which might affect an adipocyte throughout differentiation. We checked for the critical expression of PPAR family members, most prominent PPARG, in previously published RNA Seq data on SGBS adipogenesis.^31^ Compared with the initiation of differentiation, PPARG expression reached a plateau at day 4 of differentiation, similar to that of its coactivator PPARGC1B (Figure 3A). Around this time, the second wave of transcriptional cascades during adipogenesis, with its main players PPARγ and C/EBPα, allows for subsequent transcription of key adipocyte genes related to glucose and lipid metabolism.^35,36^ Lysates of differentiating SGBS cells for TPP were thereupon generated at days 4 and 12, covering an early and a terminal state of adipocyte differentiation (Figure 3B).

**Figure 3:**
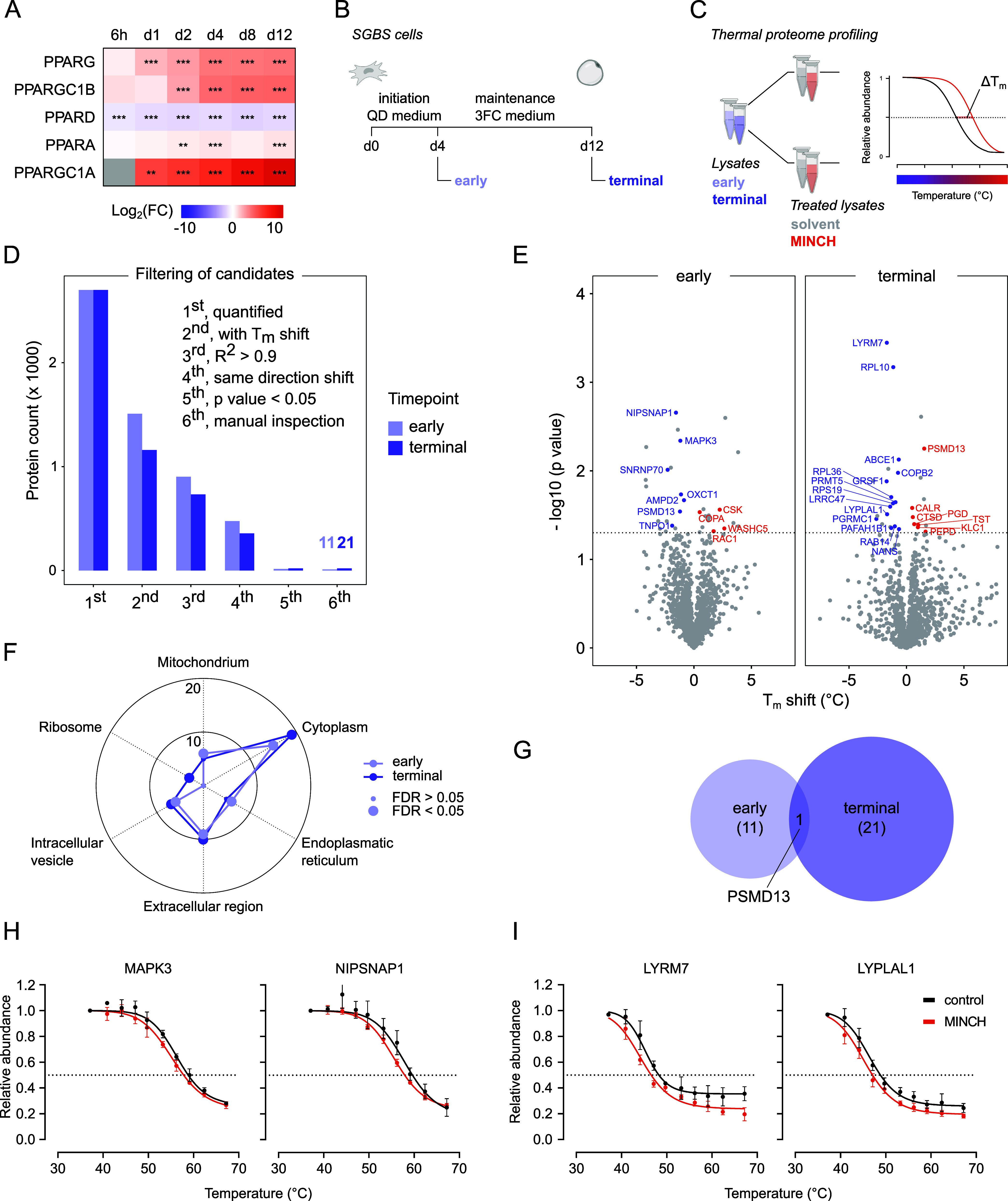
*Thermal proteome profiling* for the identification of putative interaction candidates of MINCH in SGBS cells. (A) Clustered expression of PPAR family members and their coactivators across adipogenesis of SGBS cells. Data from Aldehoff et al. 2024.^31^ (B) Experimental setup of lysate generation for subsequent *Thermal Proteome Profiling* (TPP). Lysates of SGBS cells were generated for early (d4) and terminal (d12) differentiation in *n* = 4, respectively. (C) Setup of the TPP using SGBS lysates from early and terminal differentiation, each for incubation with solvent and 10 μM MINCH. Treated lysates were exposed to a ten-step thermal gradient, and protein melting curves are displayed as relative abundance for the corresponding incubation temperature. Δ*T*_m_ indicates the MINCH-induced shift in protein melting temperature compared to the solvent control, defined as *T*_m_ (°C) at 0.5 relative abundance. (D) Candidate filtering of quantified proteins by multiple criteria resulted in 11 and 21 putative interaction candidates with MINCH in the early and terminal SGBS lysates, respectively. (E) Volcano plot indicating Δ*T*_m_ and -log_10_ (*p* values) of all quantified proteins. Candidates after manual inspection of melting curves are displayed with gene name and color (blue, negative Δ*T*_m_; red, positive Δ*T*_m_). (F) Subcellular compartment enrichment with candidate subsets early and terminal using SubcellulaRVis.^37^ (G) Overlap between the candidate sets for early and terminal differentiation. (H) Melting curves of candidates for early differentiation, MAPK3, and NIPSNAP1. (I) Candidate melting curves for terminal differentiation, LYRM7, and LYPLAL1. Solvent- and MINCH-treated protein melting curves are displayed in black and red, respectively. Data are shown as mean ± SD of *n* = 4. FC, fold change; FDR, false discovery rate; MINCH, monoisononyl-cyclohexane-1,2-dicarboxylate; SGBS, Simpson–Golabi–Behmel syndrome adipocyte cell line; Δ*T*_m_, melting point shift.

The concept of TPP emerged from cellular thermal shift assays (CETSA) in combination with high-resolution tandem mass-tagged (TMT) mass spectrometry-based proteomics, to identify protein interactors of drugs or other small molecule ligands on a proteome-wide scale.^38^ The binding of a chemical or drug to a protein is thought to change its susceptibility toward thermal denaturation (Δ*T*_m_), thereby altering its solubility at different incubation temperatures compared to a solvent-treated control (Figure 3C). The *early* and *terminal* lysates were divided and incubated with solvent or 10 μM MINCH, respectively (Figure 3C). The treated lysates were further divided for incubation along a ten-step temperature gradient, the denatured protein was removed by centrifugation, and the soluble fraction was prepared for LC-MS/MS and TMT-based protein quantification.

Quantified proteins were subjected to stringent filtering for putative MINCH protein interaction partners (Figure 3D). The final selection of candidate interactors comprised those proteins that showed a significant *T*_m_ shift of MINCH vs solvent-treated protein, indicating the same shift direction for all four replicates, with melting curve fits of *R* ^2^ > 0.9 and manually inspected melting curves (Figure 3D). The 11 and 21 candidates for the early and terminal differentiation, respectively, are displayed in the volcano plots, indicating *T*_m_ shifts and -log_10_ (*p* values) (Figure 3E). An enrichment for Gene Ontology Cellular Components (GOCC) using the tool SubcellulaRVis,^37^ indicated a comparable distribution of the candidates for early and terminal differentiation on the cellular compartments (Figure 3F). Similarly, most candidates of both data sets were cytoplasm-associated. While *early* candidates showed a slightly stronger enrichment for mitochondria and endoplasmic reticulum cellular components, *terminal* candidates included more ribosome associations (Figure 3F). Between the two sets of protein candidates, only the 26S proteasome non-ATPase regulatory subunit 13 (PSMD13) overlapped (Figure 3G).

The *early* candidate selections included candidates with kinase function (e.g., MAPK3 (Mitogen-activated protein kinase 3, also referred to as extracellular signal-regulated kinase 1 (ERK1)) and C-terminal c-Src kinase (CSK)) and active roles in mitochondrial integrity (4-nitrophenylphosphatase domain and non-neuronal SNAP25-Like 1 (NIPSNAP1); Figure 3H). Likewise, the set of *terminal* candidates included those important for mitochondrial homeostasis, function and gene expression (e.g., progesterone receptor membrane component 1 (PGRMC1), LYR motif containing 7 (LYRM7), and G-Rich RNA sequence binding factor 1 (GRSF1)) or with roles in adipocyte differentiation and associations to obesity (lysophospholipase-like 1 (LYPLAL1); Figure 3I). In particular, the activities of ERK1 and, to some extent, CSK, have been linked to insulin signaling, and disruption of these could ultimately lead to insulin resistance.^39–41^

For most interaction candidates, incubation with MINCH significantly reduced their *T*_m_ (Figures 3H,I, S3 and S4). A broader overview of candidate melting curves can be found in the Supporting Information (Figures S3 and S4). The candidate protein abundances after rosiglitazone- and MINCH-induced adipogenesis indicate a high similarity between SGBS and SVF adipocytes (Supporting Information Figure S5).

### MINCH Alters the Acetylome and Phosphoproteome in Differentiating SGBS Cells *In Vitro*

As the TPP approach revealed putative interaction partners of MINCH in differentiating SGBS cells that might impact global protein acetylation and phosphorylation by the regulation of mitochondrial gene expression and homeostasis and its kinase activities, we decided to study the acetylome and phosphoproteome under MINCH-exposed adipogenesis. Protein post-translational modifications (PTMs) like acetylation and phosphorylation are dynamically involved in adipocyte differentiation^31^ and can serve as proxies for cellular signaling. While phosphoproteomics has emerged as an established methodological approach to study overall cellular signaling in diverse disease conditions,^42,43^ the acetylome has been shown to be more closely connected to the regulation of central cellular metabolism in the mitochondria.^44,45^

To study the effect of MINCH exposure on the signatures of acetylation and phosphorylation in SGBS cells undergoing adipocyte differentiation, the cells were exposed to different combinations of differentiation agents and inhibitors for a duration of 12 days (Figure 4A). We explicitly wanted to discriminate between PPARγ-dependent and independent effects on the cell’s PTM profiles. Thus, experimental conditions included the selective and irreversible PPARγ antagonist GW9662 alone and in combination with the control differentiation agent rosiglitazone and MINCH (Figure 4A). Cell lysates were generated 12 days postadipogenic induction, and LC-MS/MS sample preparation was performed according to a sequential PTM enrichment protocol (Figure 4B). Tryptic peptides were prepared from bulk proteome samples, acetylated peptides were enriched using antiacetyl-lysine agarose beads for immunoaffinity purification, and the subsequent flow-through was further enriched for phosphopeptides using TiO_2_ and Fe-NTA (Figure 4B). These methods used to enrich phosphorylated peptides introduce a bias toward serine and threonine phosphorylation. To explicitly target tyrosine phosphorylation, different enrichment strategies are required.

**Figure 4:**
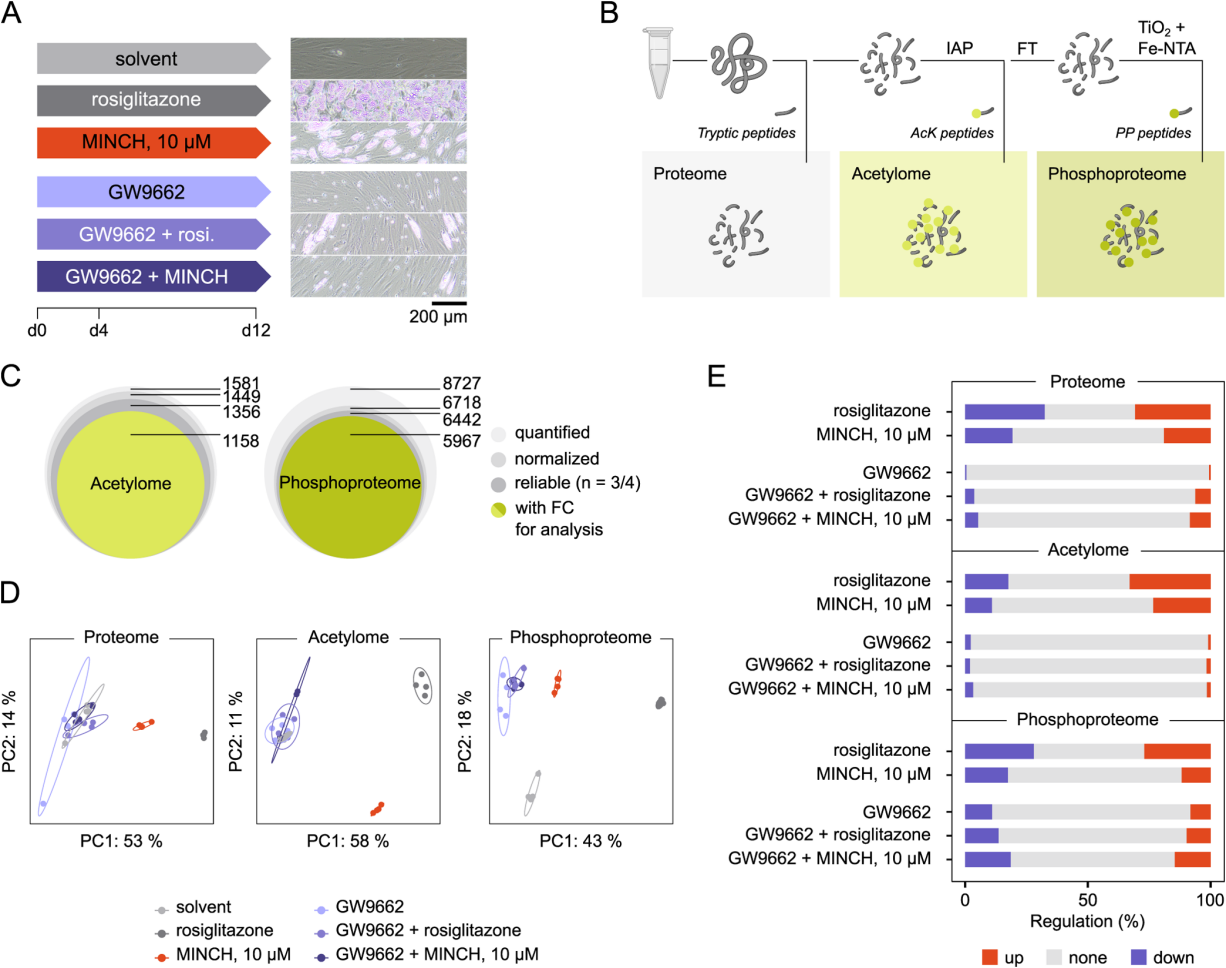
Acetylome and phosphoproteome analysis of SGBS cells after MINCH-exposed differentiation. (A) Experimental conditions include solvent and differentiation control (rosiglitazone, 2 μM), 10 μM MINCH exposure, and the respective combinations with PPARγ antagonist GW9662 (10 μM). Cells were differentiated according to the standard protocol for 12 days. At day 4, QuickDiff medium was replaced by a maintenance 3FC medium. Light microscopy images show the SGBS cells at day 12 of differentiation. The scale bar refers to 200 μm. (B) Scheme for preparation of proteome, acetylome, and phosphoproteome samples using sequential PTM enrichment techniques. (C) PTM data processing prior to differential analysis includes proteome normalization and reliability filtering. The final data set sizes of acetylome and phosphoproteome for subsequent analysis are highlighted. (D) Principal component analysis of proteome, acetylome, and phosphoproteome data for the different exposure conditions. (E) Regulation of proteome, acetylome, and phosphoproteome relative to the solvent control in response to the different exposure conditions. Experiments were performed with *n* = 4 for proteome, acetylome, and phosphoproteomes. AcK, lysine acetylation; Fe-NTA, ferric nitrilotriacetate; FC, fold change; FT, flow-through; IAP, immunoaffinity purification; MINCH, monoisononyl-cyclohexane-1,2-dicarboxylate; PP, phosphorylation; TiO_2_, titanium dioxide.

Overall, we quantified 1581 acetylated (AcK) and 8727 phosphorylated (PP) sites, 1158 (AcK) and 5967 (PP) of which were part of the protein-normalized, quality-filtered set for downstream analysis (Figure 4C). Proteome, acetylome, and phosphoproteome of rosiglitazone- and MINCH-differentiated SGBS cells were clearly separated in the PCAs (Figure 4D). The solvent control displayed a distinct profile separated from the GW9662 conditions for the phosphoproteome, while they clustered together in the proteome and acetylome (Figure 4D). Similarly, the presence of GW9662 during SGBS differentiation only rendered very few proteins and AcK-sites differentially abundant, when compared to the solvent control (Figure 4E). Yet, those conditions showed larger proportions of regulation for the phosphoproteome. Rosiglitazone and MINCH-exposed differentiating SGBS cells showed comparable proportions of regulated proteins, AcK- and PP-sites for all three data sets, while MINCH generally led to less significant changes in protein/site abundance than rosiglitazone-induced differentiation (Figure 4E).

An enrichment of pathways and terms using the databases GOBP and KEGG indicated the involvement of similar processes in proteome and acetylome data when comparing adipocytes differentiated in the presence of rosiglitazone and MINCH (Figure 5A). Positively enriched processes (median log_2_(FC) > 0) in the mentioned data sets mainly related to respiration as well as central metabolism around pyruvate, the TCA cycle, fatty acids, and lipids, while negative enrichments (median log_2_(FC) < 0) were found for chromatin organization. The presence of GW9662 in the exposure conditions clearly suppressed these pathways. The analysis of differentially phosphorylated proteins showed little treatment-specific effects on enriched pathways, yet intracellular transport, cytoskeletal organization, and ErbB and insulin signaling were affected by exposure to the differentiation agents/inhibitors (Figure 5A).

**Figure 5:**
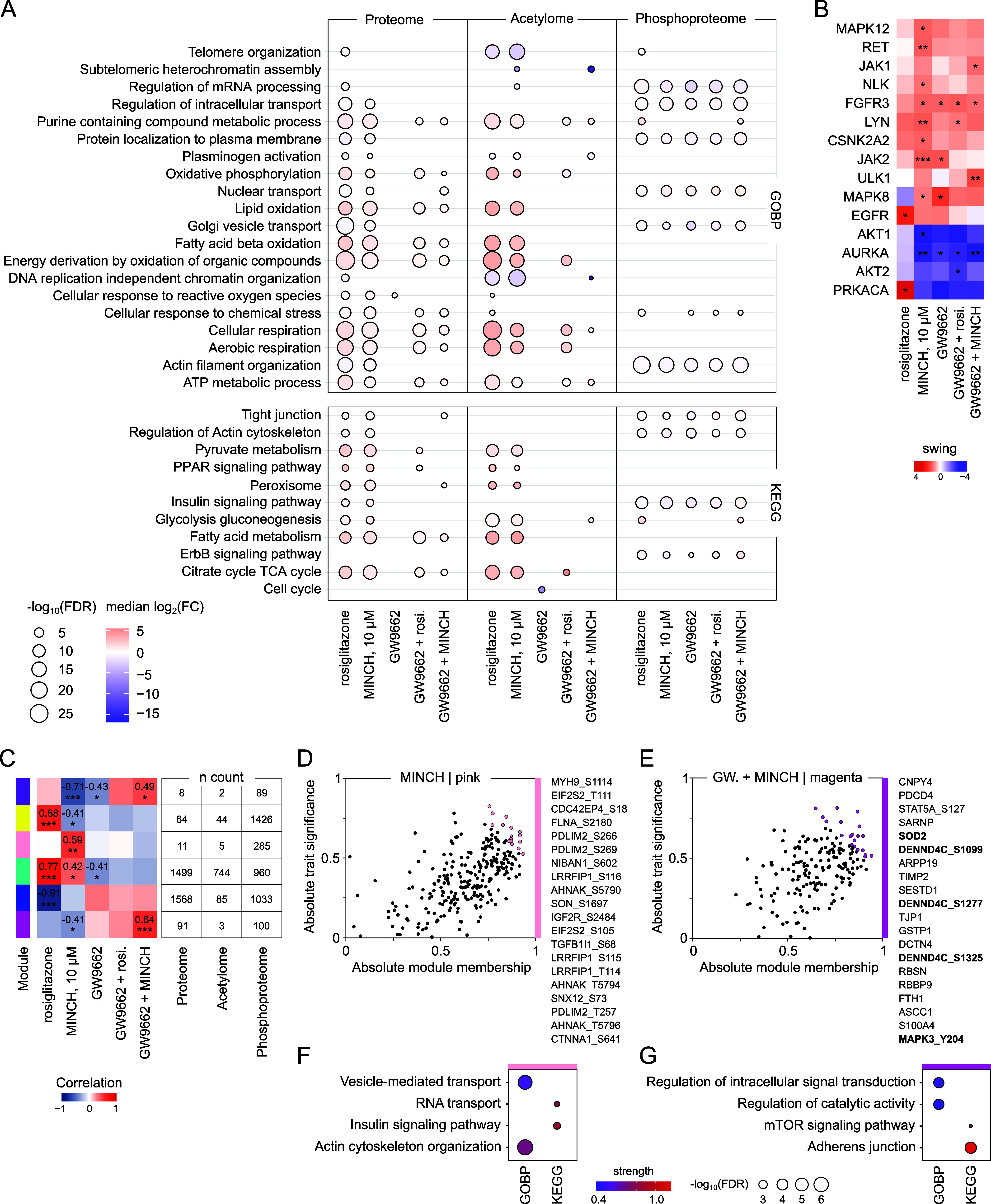
Integrated enrichment and WGCNA of proteome, acetylome, and phosphoproteome in MINCH-exposed SGBS cells. (A) Enrichment of proteome, acetylome, and phosphoproteome data using the MSigDB and GOBP and KEGG terms. Only significant pathway enrichments with FDR ≤ 0.05 are displayed as median log_2_(FC) with −log_10_(FDR) after a Fisher’s exact test. (B) Predicted kinases based on differentially phosphorylated proteins using *KinSwingR* network-based kinase activity prediction.^46^ The selection is based on relevance to metabolic regulation and the regulation of cellular stress. Indicated are swing score and corrected *p* value levels (≤0.05, *; ≤0.01, **; ≤0.001, ***). The complete list of predicted kinase activity is presented in the Supporting Information (Figure S6). (C) Module-trait-correlation matrix with the most relevant modules and their *n* count of proteins and AcK- and PP-sites. (D) Module members displayed based on their module membership and trait significance. Top 20 key drivers of the pink module for the MINCH exposure condition are highlighted in color and are listed on the right. (E) Top 20 key drivers of the magenta module for the GW9662+MINCH exposure highlighted in color and listed on the right. (F) Top 2 enriched GOBP/KEGG terms among the members of the pink and (G) magenta module with strength of enrichment and −log_10_(FDR). All data were calculated based on *n* = 4. FC, fold change; FDR, false discovery rate; GOBP, Gene Ontology Biological Processes; KEGG, Kyoto Encyclopedia of Genes and Genomes; MINCH, monoisononyl-cyclohexane-1,2-dicarboxylate.

To gain a better resolution of the effects on the phosphoproteome below the phosphoprotein level, we used the *KinSwingR* package^46^ for a network-based kinase activity prediction of involved kinases based on differentially phosphorylated sites with their sequence motifs. The activity of kinases with particular relevance for metabolic regulation and the regulation of cellular stress was found to be significantly affected by the applied differentiation conditions (Figures 5B and S6). A significant and positive swing score, indicating stronger kinase activity for the respective treatment compared to the control condition, was specific to PRKACA for rosiglitazone-conditioned differentiation. Multiple kinases, including MAPK12, RET, LYN, JAK2, and MAPK8, showed significant and positive swing scores for the MINCH condition, indicating increased activity during MINCH-conditioned differentiation (Figure 5B). A significant positive predicted activity of JAK1 and ULK1 was specific to the differentiation in the presence of GW9662 + MINCH.

The identification of key proteins, AcK- or PP-sites, could indicate central drivers for the molecular mechanisms underlying MINCH exposure during adipocyte differentiation. Thus, we performed a WGCNA, thereby integrating proteome, acetylome, and phosphoproteome data (Figure 5C). Based on the abundance profiles across conditions, proteins and sites were grouped into color-coded modules that correlated with the specific exposure conditions. The six most relevant modules included the two largest ones with positive and negative correlations for the differentiated SGBS phenotype after rosiglitazone- and MINCH-exposed differentiation (turquoise and blue) and more condition-specific correlations (Figure 5C). The pink module consisted of 11 proteins and 5 AcK- and 285 PP-sites and showed a significant positive correlation with the exposure to 10 μM MINCH. The top 20 key driver selection of this module contained only PP-sites at proteins related to RNA binding and the actin cytoskeleton (Figure 5D), which is also reflected in an enrichment of all module members using GOBP and KEGG terms (Figure 5F). Similarly, the magenta module showed the strongest positive correlation significant for GW9662+MINCH exposure (Figure 5C). It consisted of 91 proteins, 3 AcK- and 100 PP-sites, and among its members, GOBP and KEGG terms of signal transduction and adherens junctions were found enriched (Figure 5G). Its top 20 key drivers contained proteins and PP-sites, including three PP-sites at DENND4C involved in the cellular insulin response (Figure 5E).

### *In Vivo* Tissue Profiles of Phosphorylation and Acetylation of Different Adipose Tissue Depots Are Affected by Chronic Dietary DINCH Exposure in Mice

Based on the observed differences in PTM profiles between control and MINCH-differentiated adipocytes *in vitro*, the identification of stress kinases and key drivers involved in MINCH exposure, we hypothesized that dietary exposure to the parent compound and metabolic disruptor DINCH may also influence adipose tissue PTM profiles in a model of diet-induced obesity *in vivo*. The species difference between the human and murine model could, however, affect direct comparability. In 3T3-L1 adipocytes, addition of rosiglitazone (1 μM), DINCH, or MINCH (0.001–50 μM) to the differentiation medium did not cause an increase in lipid accumulation, but rather showed a weak inhibition (Supporting Information Figure S7A). Similarly, in primary murine SVF cells from subcutaneous (inguinal) or visceral (epididymal) adipose tissue of male mice, no lipid accumulation was induced through exposure to DINCH or MINCH at 0.01–10 μM (Supporting Information Figure S7B).

To target subphenotype molecular alterations in PTM profiles, we exposed 4-week-old male and female C57BL/6N mice to four different experimental diets (standard chow diet, SD; high-fat diet, HFD; HFD + low DINCH 4500 ppm, LD; HFD + high DINCH 15,000 ppm, HD) over the course of 16 weeks (Figure 6A).^47^ All mice were fed an SD during the first 4 weeks, and subsequent sample groups were allocated randomly. Body weight was not significantly affected by the subsequent dietary DINCH exposure.^47^ Visceral (VIS AT, gonadal (female), epididymal (male)) and subcutaneous (SC AT, inguinal) adipose tissues were analyzed for male (m) and female (f) mice separately. A bulk tissue analysis does not allow discrimination between the different cell types present in adipose tissue. Besides mature adipocytes, which represent the major cell type found in AT, the SVF harbors other cell types, including immune cells, fibroblasts, and preadipocytes, as well as vascular cells. It cannot be ruled out that other cell types than adipocytes contribute to the observed data. Due to the limited amount of sample input material, we pooled the lysates of 7 to 8 mice per group and analyzed the data in technical triplicate to increase PTM yield (Supporting Information Table S2).

**Figure 6:**
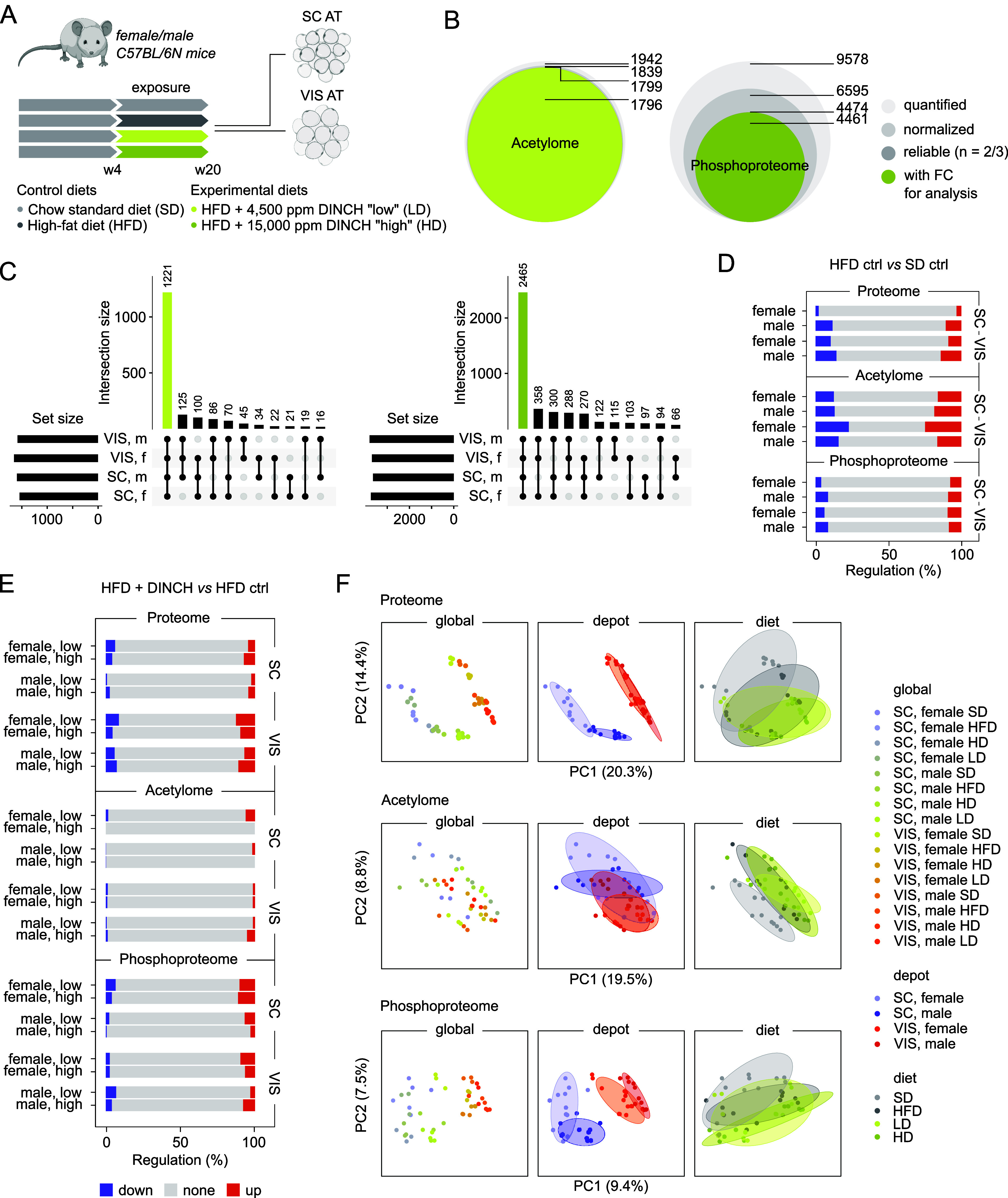
Protein and PTM signatures in visceral (gonadal/epididymal) and subcutaneous (inguinal) adipose tissue after dietary DINCH exposure in a model of diet-induced obesity. (A) Experimental setup of dietary DINCH exposure in female and male C57BL/6N mice. (B) PTM data processing of quantified sites prior to differential analysis includes proteome normalization and reliability filtering. Experiments were performed in technical triplicate. The final data set sizes of acetylome and phosphoproteome for subsequent analysis are highlighted. (C) PTM data set overlap between different sample groups for acetylome (left) and phosphoproteome (right). VIS, visceral (gonadal/epididymal) adipose tissue; SC, subcutaneous (inguinal) adipose tissue; f, female; m, male. (D) Relative regulation of the three data sets comparing tissues of mice on a standard diet with those of control high-fat diet-fed mice. (E) Relative regulation of proteome, acetylome, and phosphoproteome after dietary DINCH exposure in two different doses (low, 4500 ppm; high, 15,000 ppm). (F) Principal component analysis of the three data sets globally and in regard to adipose tissue depot and sex of the mice. DINCH, diisononyl-cyclohexane-1,2-dicarboxylate; f, female; FC, fold change; HD, HFD + 15,000 ppm DINCH “high”; HFD, high-fat diet; LD, HFD + 4500 ppm DINCH “low”; m, male; SC AT, subcutaneous/inguinal adipose tissue; SD, standard chow diet; VIS AT, visceral/epididymal adipose tissue.

Acquisition of acetylome and phosphoproteome data rendered 1942 AcK- and 9578 PP-sites across all samples, 1796 and 4461 of which were reliably identified, normalized to their corresponding protein abundance, and available for subsequent analysis of differential abundance (Figure 6B). Individual set sizes of the four different sample groups VIS/SC and m/f were similar for acetylome and phosphoproteome and showed a core of 1221 AcK- and 2465 PP-sites common to all (Figure 6C). The PCA analysis of all three data sets showed good reproducibility between replicates and a clear separation by depot origin for proteome and phosphoproteome data, with partial separation by sex (Figure 6F). The acetylome data nicely separated SD and HFD-based diets, but not clearly depot or sex. Comparing ATs from HFD- to SD-fed mice, the acetylome generally indicated the largest proportion of differentially abundant sites, especially the VIS AT of females (Figure 6D). Site regulation in the phosphoproteome appeared to be very similar in all four groups. The proteome regulation was similar between VIS groups and male-derived SC AT, except for markedly less significant alterations in the female SC AT. Dietary DINCH exposure caused low proportions of differential abundances at the protein and site levels, with the lowest amount of alterations seen in the acetylome data (Figure 6E). Proteome and phosphoproteome regulation rendered no clear trend toward exposure concentration, sex, or depot dependency.

An enrichment of proteome, acetylome, and phosphoproteome data using GOBP and KEGG terms indicated increased glycolysis/gluconeogenesis and central metabolism at the protein level, increased abundance of acetylation of central metabolic enzymes, while reduced acetylation of PPAR signaling-involved enzymes, and cytoskeleton-related protein phosphorylation, similarly for VIS and SC AT of HFD—compared to SD-fed mice (Supporting Information Figure S8–S10).

The proteome of DINCH-containing HFD-fed mice was positively enriched for pathway terms associated with respiration and core metabolic processes (glycolysis/gluconeogenesis, TCA cycle), including those related to fatty acid, lipid metabolism, and PPAR signaling (Figure 7A). Generally, there was only a minor concentration dependency for the administered low vs high DINCH concentration but a tendency toward more pronounced effects in the proteome of VIS AT of male and female mice. The effects seen for differentially acetylated proteins predominantly corresponded to the effects on core and fatty acid metabolism and respiration, as also seen for the global proteome. Beyond that, differentially phosphorylated proteins indicated an enrichment for the regulation of cell structural organization, adhesion, and protein polymerization as well as on glycolysis/gluconeogenesis and insulin signaling. Acetylome- and phosphoproteome-enriched terms displayed higher median log_2_(FC) compared to the proteome data but less consistency across groups and administered DINCH concentrations (Figure 7A). The dominant correlation in the integrated data on proteome, acetylome, and phosphoproteome was found for the two different depots, subcutaneous and visceral, across all treatment conditions (Supporting Information Figure S11).

**Figure 7:**
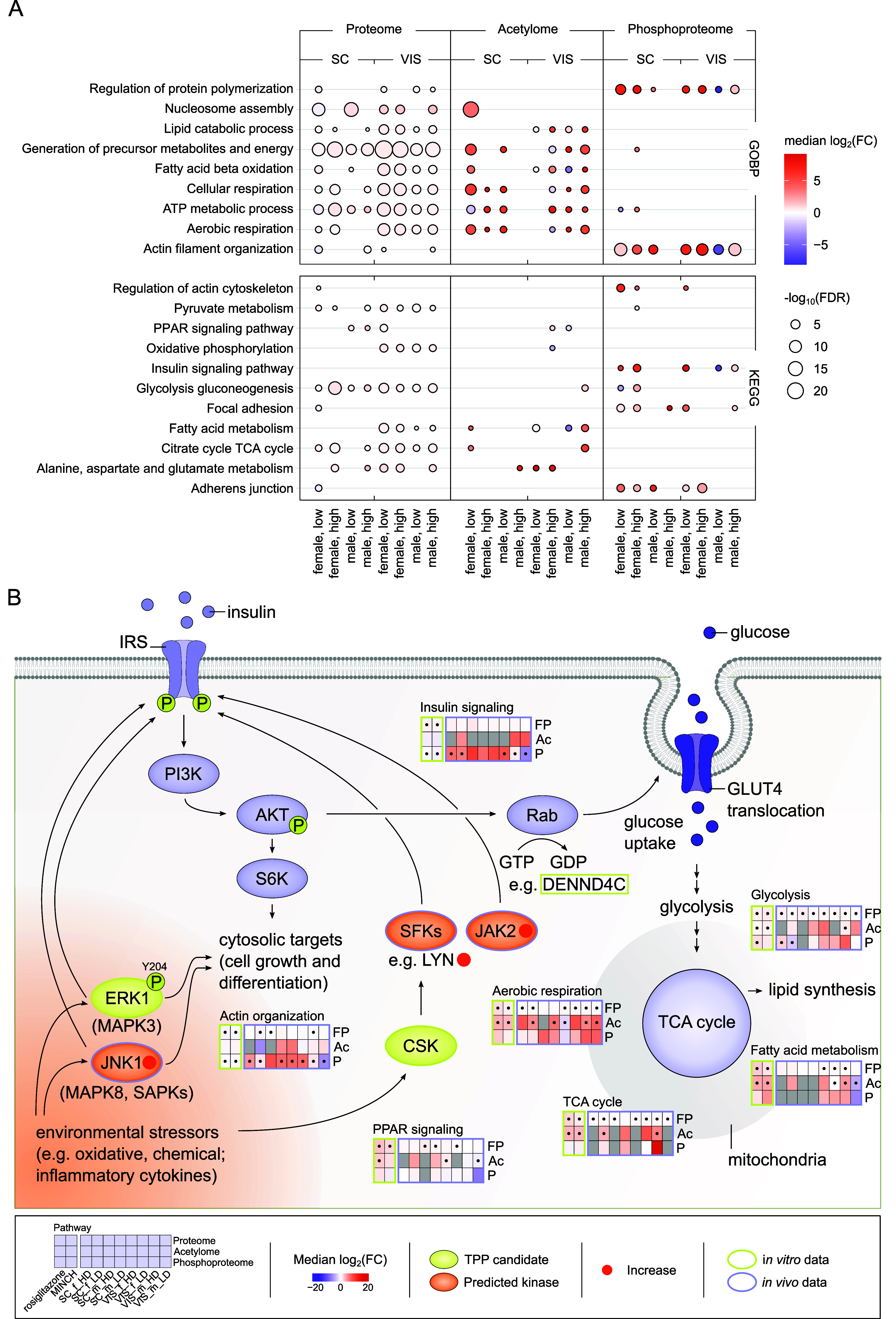
Enrichment and comparative analysis of adipose tissue proteome, acetylome, and phosphoproteome following dietary DINCH exposure. (A) Enrichment of proteome, acetylome, and phosphoproteome data using the MSigDB and GOBP and KEGG terms. Only significant pathway enrichments with FDR <0.05 are displayed as median log_2_(FC) with −log_10_(FDR) after a Fisher’s exact test. Dietary DINCH exposure in combination with HFD in two different doses: low, 4500 ppm; high, 15,000 ppm. (B) Overview on key findings of the study with a focus on insulin signaling and central metabolism. The general scheme of insulin signaling was used as a backbone. Shown is the interplay of TPP interaction candidates, predicted kinases and enriched pathways for full proteome (FP), acetylome (Ac), and phosphoproteome (P) data as median log_2_(FC) relative to the solvent control (rosiglitazone and MINCH) and the HFD control (SC and VIS tissue data), respectively, with significance indicated as *p* values <0.05, •. Included is data from the analysis of MINCH on SGBS cells (rosiglitazone, MINCH) and tissue data from SC and VIS AT of dietary DINCH (4500 ppm, LD; 15,000 ppm, HD) exposed female and male mice. The *in vitro* and *in vivo* origin of the presented data is indicated, respectively. Ac, acetylation; AKT, protein kinase B; CSK, C-terminal Src kinase; ERK1, extracellular signal-regulated kinases, also MAPK1, mitogen-activated protein kinase 1; FC, fold change; FDR, false discovery rate; FP, full/global proteome; GLUT4, glucose transporter 4; IRS, insulin receptor substrate; JAK2, Janus kinase 2; JNK1/MAPK8, c-Jun N-terminal kinases 1; PI3K, phosphoinositide 3-kinase; P, phosphorylation; S6K, S6 kinases; SAPKs, stress-activated protein kinases; SC, subcutaneous/inguinal adipose tissue; SFKs, Src family kinases; VIS, visceral/epididymal adipose tissue.

In combination, DINCH and/or MINCH exposure or its proteome-wide interaction indicates an involvement of kinases in its mode of action, thereby affecting the phosphoproteome in exposed differentiating SGBS cells and adipose tissue of exposed mice in their insulin signaling and actin organization (Figure 7B). Further downstream, effects are most prominent for the proteome and acetylome on central metabolic pathways of the *in vitro* and *in vivo* models studied here (Figure 7B).

## Discussion

Manufactured chemicals are present in all domains of life. The production of anthropogenic chemicals has by now reached the capacity of regulators to assess their risk, and exceeds the safe operating space of the planetary boundary of “novel entities.”^48^ Consequently, risk assessment strategies need to be adapted to facilitate and improve the assessment of chemical safety, ensure appropriate regulation, and protect both environmental and human health. Adipose tissue health can be impacted by exposure to chemicals with endocrine or metabolism-disrupting properties.^3^ While functional adipose tissue is a highly plastic organ that is critical for the adaptation to whole-body energy needs and glucose uptake through insulin and endocrine signaling, its dysfunction can have profound consequences for various diseases, including metabolic and cardiovascular diseases.^49,50^ Given the ever-increasing number of people living with obesity and associated health consequences in societies worldwide, this link needs to be taken seriously to develop prevention strategies.

In this study, we demonstrate the utility of advanced proteomics approaches as omics-based NAMs to enrich the regulatory ground for informed and safe regulation of chemicals with potentially metabolism-disruptive properties, exemplified by DINCH. Hexamoll DINCH entered the European market in 2002 as a replacement for DEHP, especially in sensitive applications.^51^ In the following years, restrictions forced back the use of DEHP, thereby increasing the importance of DINCH as a substitute.^52–55^ Concerns about the safe use of DINCH have been raised as a result of its increasing prevalence in diverse population matrices^56,57^ and the potential of its primary metabolite MINCH to initiate adipocyte differentiation in rat SVF^58^ and human SGBS cells.^17^ We observed MINCH exposure at 10 μM to initiate adipocyte differentiation and lipid accumulation in human subcutaneous adipose tissue SVF cells (Figures 1 and 2), confirming the results of the aforementioned study in human SGBS cells,^17^ and supporting the robustness, usefulness, and validity of the SGBS adipocyte model.^17,18^ Although SGBS cells display a specific genetic background^59^ and lack donor variability in terms of sex and bodily origin, both human adipocyte cell models allowed the same conclusions to be drawn. This led us to conclude that the use of SGBS cells is informative, and we continued to further explore the adversity of DINCH/MINCH exposure in this model.

Beyond the assumed mode of action via interaction of MINCH with PPARγ, leading to the expression and translation of adipogenic markers and ultimately adipocyte differentiation, MINCH likely displays further routes of action through other interactors. Commonly, drugs or small molecules interact with ∼11 more proteins than just their intended target, enabling alternative modes of action and potential off-target effects.^33,34^ Here, we identified potential MINCH-interaction partners using *thermal proteome profiling* (TPP) as a discovery approach, considering the complete SGBS proteome. TPP has emerged from cellular thermal shift assays (CETSA) that have been combined with tandem mass tag (TMT) mass spectrometry-based proteomics.^38^ An observed shift in a protein’s melting temperature is hereby interpreted as a ligand-induced change in the protein’s conformation and, thus, altered susceptibility to thermal denaturation. A similar methodological approach, namely, 2D proteome integral solubility alteration (2D PISA), has facilitated the identification of molecular initiating events (MIE) in 2,3,7,8-tetrachlorodibenzo-*p*-dioxin (TCDD) exposed HepG2 cells, aiming to contribute to the development of adverse outcome pathways (AOPs).^60^ For the SGBS proteome, we identified putative MINCH-interaction candidates with kinase function and those involved in the maintenance of mitochondrial integrity, adipocyte differentiation, and associations to obesity. One candidate, PSMD13, was identified in both time points analyzed. PPARγ, as a nuclear receptor thought to decisively mediate the adipogenic effect of MINCH, was not found among the interaction candidates, as the resolution of bulk proteomics approaches typically does not allow for the identification of most nuclear receptors or transcription factors.

Extracellular signal-regulated kinase 1 (ERK1), also referred to by its encoding gene name MAPK3, has been identified as a potential MINCH interaction partner during early adipogenesis by a negative *T*_m_ shift upon MINCH incubation (*early*, Figure 3E). This is thought to indicate an indirect interaction where the presence of MINCH is affecting the overall state of the protein, in contrast to a positive Δ*T*_m_ induced by direct compound binding.^38^ ERK was found important in differentiating murine Ob1771 and 3T3-L1 adipocytes with a temporally distinct role in the early onset of adipogenesis.^61,62^ Deficiency of ERK1 in mice resulted in a lower number of adipocytes compared with wild-type animals. These ERK1^–/–^ mice were not prone to HFD-induced obesity and insulin resistance^63^ or at least showed marked metabolic improvements without effects on adiposity, when inbred with leptin-deficient ob/ob mice (ob/ob ERK1^–/–^).^41^ Similarly, ERK1/2 activity was found to be increased during 3T3-L1 adipocyte hypertrophy and in two *in vivo* models of type 2 diabetes,^39^ as well as in human adipose tissue of individuals with diabetes.^64,65^ While an inhibition of mitogen-activated protein kinase kinase (MEK) upstream of ERK signaling improved diabetic parameters and adipocytokine profiles in hypertrophic 3T3-L1 cells,^39^ treatment with adipogenic hormones, including dexamethasone, IBMX, insulin, and FGF-2, led to a stimulation of MEK/ERK activity.^62^ Insulin resistance is a major risk factor for the development of type 2 diabetes and is significantly associated with abdominal obesity. Considering that an explicit role of ERK1 has been demonstrated during early adipogenesis, where we identified MINCH as an indirect interaction partner, a suggested role of MINCH in altering the physiological state of ERK1 could influence target protein phosphorylation and insulin signaling with potential downstream implications for insulin resistance.

Another kinase identified as a potential MINCH interaction partner is the C-terminal c-Src kinase (CSK; *early*, Figure 3E). It signals upstream of ERK1 by negatively regulating Src family kinases, and is thought to modulate c-Src to inactivation through insulin-like growth factor 1 (IGF-I) stimulation in differentiating 3T3-L1 adipocytes.^40^ CSK has also been mentioned as a candidate gene in a genome-wide association study (GWAS), where its expressing allele was increased in obesity, while suggesting favorable for cardiovascular health, with no further biological evidence just yet.^66^ Still, both ERK1 and CSK are involved or associated with insulin signaling through the translation of external stimuli by target phosphorylation, underlining MINCH’s potential to affect adipose tissue health.

A different mode of action is indicated by the putative MINCH interaction candidate 4-nitrophenylphosphatase domain and non-neuronal SNAP25-Like 1 (NIPSNAP1) (*early*, Figure 3E). NIPSNAP1 is a mitochondrial matrix protein involved in autophagy receptor recruitment for subsequent autophagy of depolarized mitochondria (mitophagy).^67^ Additionally, it negatively regulates oxidative stress-induced cellular senescence in HCT116 and HepG2 cells through a mechanism dependent on superoxide dismutase 2 (SOD2) activation^68^ and is shown to be important in maintaining an elevated energy expenditure in brown adipose tissue (BAT) during nonshivering thermogenesis (NST).^69^ Oxidative stress and mitochondrial damage are well-known effects of exposure to diverse chemicals with ubiquitous environmental presence, among them legacy phthalates like DEHP and its metabolite MEHP.^70,71^ The consequences of oxidative stress and mitochondrial damage can be profound for cardiovascular^72,73^ and metabolic diseases,^74^ as well as chronic inflammation.^75^ A putative indirect interaction of MINCH with NIPSNAP1 (-Δ*T*_m_) could possibly affect its role in the effective clearance of damaged mitochondria or maintenance of energy metabolism with potential subsequent health effects. Generally, a TPP approach using living cells,^38^ which is so far not feasible for mature adipocytes due to their extensive lipid load and resulting fragility, could increase cellular plausibility of the identified interaction candidates in other cell models.

Beyond the indication of potential protein interaction candidates of MINCH in differentiating SGBS cells by TPP, global proteome data provide close to phenotype information that can be further enriched with data on the profiles of protein post-translational modifications, such as acetylation and phosphorylation. This allows a refined understanding of cellular signaling within cells and tissues and potentially points to actively important signaling events, pathways, and relations between proteins.

Apart from the observed changes in the proteome of SVF and SGBS cells as well as visceral (gonadal/epididymal) and subcutaneous (inguinal) adipose tissue following the exposure to DINCH and its metabolite MINCH *in vitro* and *in vivo*, respectively, the profiles of acetylation and phosphorylation were affected by chemical exposure. Exposure to MINCH in SGBS cells *in vitro* resulted in a majority of observable effects mediated through PPARα and γ, as a coexposure with their selective PPAR inhibitor GW9662 did not reveal profound PPAR-independent effects for proteome and acetylome (Figure 4D). Differently, the phosphoproteome of GW9662 coexposed SGBS adipocytes indicated alterations specific for MINCH exposure independent of GW9662-mediated inhibition of PPARs (Figures 4E and 5). The pink and magenta modules of an integrative WGCNA of proteome, acetylome, and phosphoproteome showed specific and significant positive correlations for MINCH exposure in the absence and presence of GW9662, respectively (Figure 5D). Interestingly, among the top 20 key drivers of the magenta module, positively correlating with MINCH and GW9662 coexposure specifically, we found candidates previously discussed in the context of putative MINCH protein interaction partners from our TPP analysis. Among them were PP-site ERK1/MAPK3_Y204 and antioxidant protein SOD2, whose great relevance for insulin signaling integrity and the elimination of ROS during oxidative stress has been highlighted above. ERK1_Y204 and _T202 phosphorylation by MEK activates ERK1 for downstream signaling.^76^ In heart tissue and epididymal white adipose tissue (WAT), ERK1 phosphorylation at its activating residues was increased through stimulation with an insulin sensitivity promoting angiotensin II type 2 (AT_2_) agonist.^77^ In addition, three PP-sites (S1099, S1277, and S1325) of the DENN Domain Containing protein 4C (DENND4C) were found among the magenta module’s key driver candidates. DENND4C is a Rab10 guanosine exchange factor (GEF) that is important for insulin-stimulated glucose transport through GLUT4 translocation. Upon insulin stimulation of 3T3-L1 adipocytes, phosphorylation of S1043, S1096, and S1321 was found to increase, possibly mediated through AKT.^78,79^ This provides further evidence for an effect of MINCH on ERK1 activity, oxidative stress response, and insulin-mediated signal transduction for glucose transport.

In adipose tissues from *in vivo* dietary DINCH-exposed mice, the phosphoproteome appears more closely connected to the effect of DINCH than to the acetylome. In contrast, the acetylome seemed closely linked to the feed-state of the mice (SD/HFD) through direct connections to glucose and acetyl-CoA availability as well as sirtuin activation.^80^ An additional effect of chemical exposure was limited to a few changes in central and fatty acid metabolism and respiration for the time point analyzed in our setup (16-week exposure; Figure 6E). This also confirms that an effect of DINCH on protein acetylation profiles might be partly masked by the dominant effect of the HFD in comparison to SD. In this regard, it would be very interesting to study the effect of dietary DINCH exposure in an SD background, which was, however, beyond the scope of this study. Additionally, sex-dependent effects transmitted via estrogen and androgen hormonal receptors should be kept in mind for the assessment of DINCH-associated effects in future studies. Initial evidence suggests a selective activation of estrogen and androgen receptors by DINCH metabolites, thereby suggesting a potential adverse effect related to sex.^81^

Despite the known difference in the potential of environmental chemicals to modulate and activate human and murine PPARγ,^82^ other modes of chemical action mediated by DINCH/MINCH might be more conserved between both species. This aligns with the finding that human and murine models showed different responses to MINCH exposure *in vitro* regarding the initiation of adipocyte differentiation and lipid accumulation, while the results regarding enriched pathways in the proteome, phosphoproteome, and acetylome observed in MINCH-exposed SGBS cells generally aligned with the PTM signatures found in adipose tissues of orally DINCH-exposed mice. The pathway involvement and importance of PTMs for certain processes were very much in agreement. Thus, we believe that the generated *in vivo* data confirms the trustworthiness of the SGBS cells as an *in vitro* model and NAM. What appears common to all approaches undertaken in this study is the apparent link to a potential disruption of insulin signaling and central metabolism as a consequence of DINCH/MINCH exposure (Figure 7B). As mentioned earlier, a dysregulation of insulin signaling could have severe consequences for adipose tissue and, thus, metabolic health, highlighting the urge to consider DINCH as an MDC. We believe that the generated multifaceted PTM and global proteome data highlight the potential of proteomics approaches to add relevant information on the safety of chemicals by using *in vitro* models in combination with omics-based NAMs.

## Experimental Procedures

A list of all resources used can be found in the Supporting Information, Table S1.

### SGBS Cell Culture

The laboratory of Prof. Wabitsch at the University Hospital Ulm contributed Simpson–Golabi–Behmel syndrome (SGBS) cells. SGBS cells for subsequent *thermal proteome profiling* were differentiated and maintained according to the published standard protocol.^59,83^ Briefly, SGBS preadipocytes were grown to full confluency in basal culture medium, consisting of supplemented Dulbecco’s modified Eagle’s F12 (DMEM/F12; Gibco; supplements: 33 μM biotin (Sigma-Aldrich), 17 μM panthothenate (Sigma-Aldrich), 100 U/l penicillin/streptomycin (Sigma-Aldrich), and 10% fetal calf serum (FCS; Gibco)). Initiation (day 0 to 4) and maintenance (day 4 to 12) of differentiation was achieved by an exchange to complemented serum-free basal medium (supplements: 0.1 μM cortisol, 0.01 mg/mL apo-transferrin, 0.2 nM triiodothyronine, 20 nM insulin), which, specifically for the first 4 days, contained 2 μM rosiglitazone, 25 nM dexamethasone, and 200 μM 3-isobutyl-1-methylxanthine (all supplements purchased from Sigma-Aldrich) in addition. The medium was exchanged every 4 days.

For the chemical-exposed cell culture, medium supplemented with the respective treatment (solvent, 2 μM rosiglitazone (d0–4 only), 10 μM monoisononyl-cyclohexane-1,2-dicarboxylic acid ester (MINCH, purity >95%, Toronto Research Chemicals, Canada)) was exchanged every 2 days to ensure consistent exposure conditions. Conditions, including the PPARγ inhibitor GW9662 (Cayman Chemical Company), were exposed to the inhibitor 1 h prior to chemical exposure, which was only added to the culture media at 10 μM thereafter. Cells were maintained under 5% CO_2_ at 37 °C and 95% humidity.

### Human Subcutaneous Adipose Tissue Collection, Isolation of the Stromal Vascular Fraction, and Differentiation of Primary Adipocytes

Subcutaneous adipose tissue (SCAT) samples were collected during elective aesthetic and postbariatric surgery at the Division of Plastic, Aesthetic and Special Hand Surgery of the University Hospital Leipzig between January and July 2024. Written informed consent was obtained from all patients, and the study was approved by the Ethics Committee of the University of Leipzig (approval numbers: 159–12–21052012 and 017–12ek). Collection sites were (1) the lower abdomen during abdominoplasty as well as (2) the thighs during thigh lift, and (3) the back during lifting surgery. All operations were performed under general anesthesia. Electrocautery was used to prepare the subcutaneous tissue for resection. Thermally damaged tissue and skin were removed using scissors or a scalpel, and fat samples were placed into sterile sample containers for immediate processing.

Adipose tissue (AT) samples were washed, connective tissue was removed, and the fat was further processed with scissors to obtain a homogeneous mixture that was digested with 500 units collagenase type II (Gibco) per gram of tissue in adipocyte isolation buffer (100 mM HEPES, 123 mM NaCl, 5 mM KCl, 1.3 mM CaCl_2_, 5 mM Glucose, 1% ZellShield (Minerva biolabs, Germany), 4% BSA) for 45 min at 37 °C. Digested fat was passed through a 300 μm syringe strainer (pluriSelect, Germany). By insertion of a needle with a syringe into the tube, stromal vascular fraction (SVF) cells were transferred.

Cells were washed with buffer twice, followed by a red blood cell lysis using red blood cell lysis buffer (0.154 M NH_4_Cl, 0.01 M KHCO_3_, 0.1 mM EDTA) for 7 min at RT, before addition of 10 mL of DMEM/F12, followed by a further 5 min of incubation. The suspension was passed through a 30 μm MACS SmartStrainer (Miltenyi Biotec, Germany), and preadipocytes were pelleted by centrifugation at 500*g* for 10 min, followed by resuspension in growth medium (DMEM/F12 + 10% FCS + 1% Zellshield). Cells were seeded in 24-well plates at a density of 1,50,000 cells per well. Preadipocytes were cultivated in growth medium until confluency (day 0) and, subsequently, differentiation was induced by changing to induction medium from day 0–3 with either rosiglitazone (1 μM), MINCH and DINCH (10 nM and 10 μM each; MINCH, purity >95%, Toronto Research Chemicals, Canada; DINCH, purity 98%, abcr GmbH, Germany) or solvent control (Methanol/DMSO). From days 3–12, the cells were further differentiated with the differentiation medium, with medium changes every 48 h.

### Animal Husbandry, *In Vivo* Experiments, and Primary Murine Adipocyte Cultures

For primary murine adipocyte cultures, SVF from visceral (epididymal) and subcutaneous (inguinal) adipose depots of 8–12-week-old male mice (C57BL/6N) were dissected, isolated, and differentiated for 8 days, as previously described.^84^ In brief, pooled tissue pieces were homogenized and digested in HEPES isolation buffer (0.1 M HEPES, 123 mM NaCl, 5 mM KCl, 1.3 mM CaCl_2_, 5 mM glucose, 4% BSA, 1% penicillin/streptomycin, and 0.2% (w/v) collagenase II, pH 7.2) for 30 min at 37 °C. The cell suspension was passed through a 100 μm nylon filter and was subsequently incubated on ice for 15 min to let the mature adipocytes float up. SVF and mature adipocytes were separated, and the SVF fraction was passed through a 40 μm nylon filter, followed by centrifugation (700*g*, 10 min, 4 °C). The medium was removed, and preadipocytes were incubated in erythrocyte lysis buffer for 5 min. After centrifugation, cells were suspended in culture medium (DMEM containing 10% FCS, 1% penicillin/streptomycin, and 25 μg/mL sodium ascorbate), seeded, and maintained at 37 °C and 5% CO_2_. After the cells reached confluency (day 0), the medium was changed to the differentiation cocktail (culture medium supplemented with 3 nM insulin, 1 nM T3, 1 μM rosiglitazone (excluded for the conditions without rosiglitazone), 0.4 μg/mL dexamethasone, and 0.5 mM IBMX) for 2 days, followed by 2 days with 3 nM insulin in culture medium and followed by terminal differentiation in culture medium only. Chemicals (1 μM DINCH, 10 nM MINCH, 10 μM MINCH) were added to the medium under the respective conditions throughout the whole duration of differentiation.

For the feeding experiment *in vivo*, male and female C57BL/6N mice (RRID:IMSR_TAC:B6) were kept under pathogen-free conditions, maintained at 23 °C with a 12 h light/dark cycle. Four-week-old mice were provided either a standard chow diet (SD; 9 kJ% fat, V1534, Ssniff, Germany) or a high-fat diet (HFD; 59 kJ% fat, E15772–34, Ssniff, Germany) as control, or HFD supplemented with 4500 (LD) or 15,000 ppm (HD) 1,2-cyclohexanedicarboxylic acid diisononyl ester (DINCH, purity 95%, abcr GmbH, Germany), for a duration of 16 weeks. Mice had *ad libitum* access to water and food. Animal experiments were performed in compliance with the guidelines approved by the local authorities of the State of Saxony, Germany, TVV38/20. Tissues were prepared and snap-frozen in liquid nitrogen before being stored at −80 °C.

### Quantification of Lipid Accumulation

At terminal differentiation, the cells’ lipid accumulation was quantified using AdipoRed staining.^85^ The cells were washed with 200 μL of PBS once, and 200 μL of PBS and 5 μL of AdipoRed Assay Reagent (Lonza, Switzerland) were added per well. After 10 min of incubation at RT protected from light, fluorescence with excitation at 485 nm and emission at 572 nm was determined with five measurements per well using a fluorimeter.

### Cell and Tissue Lysis

For the analysis of proteome, acetylome, and phosphoproteome, differentiated SGBS cells (d12) were washed with PBS twice and harvested using 1 mL of lysis buffer (20 mM HEPES pH 8.0 (Roth, Germany), 9 M urea (Merck, Germany), 1 mM sodium orthovanadate (Sigma-Aldrich), 2.5 mM sodium pyrophosphate (Sigma-Aldrich), and 1 mM β-glycerophosphate (Alfa Aesar)). The samples were incubated for 15 min, frozen at −80 °C, and thawed on ice, and protein concentration was determined using the Pierce 660 nm protein assay (Thermo Fisher Scientific).

For cell lysis of SVF cells, the medium was removed, cells were washed with PBS twice, and lysed using 100 μL of lysis buffer (500 mM Tris HCl, 150 mM NaCl, 0.1% SDS, 0.5% sodium deoxycholate, 1% Triton X-100, 1× cOmplete protease inhibitor (Roche)). Cell lysates were incubated on ice for 1 h and centrifuged at 16,000*g* and 4 °C for 15 min, and the cleared lysates were transferred to new tubes. Protein concentrations were determined using the detergent-compatible DC Protein Assay (BioRad).

Subcutaneous (inguinal) and visceral (gonadal (female), epididymal (male)) adipose tissue from mice was lysed in urea buffer (as above). For 10 mg of the tissue sample, 50 μL of lysis buffer was used. The tissues were lysed in a precooled tissue lyser (Qiagen, Germany) at a frequency of 30 ms for 5 min. Samples were incubated for 30 min, and cell debris was collected by centrifugation at 16,000*g* at 10 °C for 15 min. The supernatant was transferred to new tubes, and the protein concentration was determined using the Pierce 660 nm protein assay as described above.

### Immunoaffinity Purification of Acetylated Lysines

Immunoaffinity purification (IAP) of acetylated lysines was facilitated by the PTMScan Acetyl-Lysine Motif [Ac–K] Kit (Cell Signaling Technology) according to the manufacturer’s protocol with slight adaptations. As input material was limited, tissue lysates were pooled condition-wise and analyzed in technical triplicate. 1 mg of protein from SGBS cell lysates and 0.48 mg of protein from tissue lysates were reduced with 5 mM dithiothreitol (DTT; GE Healthcare) for 1 h and alkylated with 10 mM iodacetamid (IAA; Merck, Germany) for 30 min before the urea concentration was diluted to <2 M with 20 mM HEPES pH 8.0. For tryptic digestion, trypsin (Promega) was added in a 1:50 (enzyme/protein) ratio and incubated at 37 °C overnight. The samples were acidified to 0.1% formic acid (FA; Fluka Honeywell), and fatty acids were precipitated by incubation for 15 min on ice and removed by centrifugation at 4000*g* for 15 min. Cleared peptide solution was desalted on Oasis 1 cm^3^ 30 mg HLB (Waters) cartridges. Briefly, columns were prepared with decreasing concentrations of acidic acetonitrile (ACN; Roth, Germany; 100%, 40%, 3% ACN in 0.1% FA); peptide solution was applied to the columns and washed with 3% ACN in 0.1% FA twice. Bound peptides were eluted with 40% acidified ACN solution, and eluates were frozen at −80 °C for 1 h to prepare for lyophilization (α 2–4 LSC, Christ, Germany). Peptides were lyophilized to complete dryness and reconstituted in the immunoaffinity purification buffer provided by the kit. The peptide solution was cleared by centrifugation at 10.000*g* for 15 min at 4 °C and incubated with a quarter of a vial of antibody beads on a rotator at 4 °C overnight. Antibody beads were collected by centrifugation, and unbound peptide was transferred to new tubes for subsequent preparation of the complementary proteome. Bound peptides were washed in IAP buffer twice and MS-grade water three times, and the fraction of acetylated peptide was eluted in 0.15% trifluoroacetic acid (TFA; Merck, Germany). Enriched acetyl-peptides were lyophilized as described above and reconstituted in 0.1% FA for MS analysis.

20 μg of protein lysates were reduced, alkylated, and enzymatically cleaved for the analysis of the complementary full proteome as described above, using a paramagnetic bead approach (Cytiva).^86^

### Phosphopeptide Enrichment

The phosphopeptides were successively enriched by using TiO_2_- and Fe-NTA-based metal affinity chromatography. Enrichment was performed on the flow-through of the antiacetyl-lysine IAP. The flow-through samples were desalted as described above, fully lyophilized, and reconstituted in phosphopeptide binding buffer. First, phosphopeptide enrichment was performed using the High-Select TiO_2_ Phosphopeptide Enrichment Kit (Thermo Fisher Scientific) according to the manufacturer’s instructions. Unbound peptides were dried and reconstituted for further enrichment. For the second enrichment step, the High-Select Fe-NTA Phosphopeptide Enrichment Kit (Thermo Fisher Scientific) was used according to the manufacturer’s instructions. The enriched phosphopeptide eluates from both steps were combined, dried, and reconstituted in 0.1% FA for MS analysis.

### Thermal Proteome Profiling

SGBS cells were differentiated into early (d4) and terminally differentiated (d12) adipocytes using the standard differentiation protocol described above. Samples were harvested using PBS complemented with 1× cOmplete protease inhibitor (Roche, Switzerland) to scrape the cells off the culture plate before snap-freezing in liquid nitrogen. The freeze–thaw cycle was repeated twice. Cell debris was removed by centrifugation, and cleared lysates were transferred to new tubes. Protein concentrations were determined using the Pierce 660 nm protein assay reagent (Thermo Fisher Scientific). Lysates were normalized to an equal protein concentration, supplemented with the respective treatment (solvent or 10 μM MINCH), and incubated at RT for 10 min. Incubated lysates were divided into 10 tubes, subjected to temperature incubation (37.1, 40.9, 44.1, 47.1, 49.7, 53.2, 56.2, 59.1, 62.4, 67.2 °C) in a thermocycler for 3 min, and were left to cool at RT for 3 min before they were snap-frozen in liquid nitrogen. Samples were thawed on ice and centrifuged at 16.000*g* and 4 °C for 30 min before the soluble fraction was transferred to new tubes. The protein concentration of the lowest incubation temperature was determined using the Pierce 660 nm protein assay (Thermo Fisher Scientific) as described above, and the volume of lysate used for subsequent sample preparation was chosen accordingly.

Samples were prepared for MS analysis using paramagnetic beads (Cytiva).^86^ In brief, proteins were reduced using 20 mM TCEP at 55 °C for 1 h, alkylated with 37.5 mM IAA at RT for 30 min, and cleaved into tryptic peptides at 37 °C overnight. Tryptic peptides were incubated with the respective tandem mass tag (TMT10plex) labels (Thermo Fisher Scientific) for 1 h at RT, the reaction was quenched using 5% hydroxylamine (Thermo Fisher Scientific), and the samples were combined. The labeled peptides were cleaned, eluted as a single fraction using ddH_2_O containing 2% DMSO, dried, and reconstituted in 0.1% FA.

### Mass Spectrometry Peptide Data Acquisition

All label-free SGBS adipocyte samples were analyzed in data-dependent acquisition (DDA) on a setup consisting of an Ultimate 3000 RS nano ultraperformance liquid chromatography system (Thermo Fisher Scientific) coupled to a Q Exactive HF Hybrid Quadrupole Orbitrap mass spectrometer (Thermo Fisher Scientific) equipped with a TriVersa NanoMate system (Advion). Peptides were trapped on an Acclaim PepMap 100 C18 column, nanoViper, 3 μm, 75 μm × 2 cm column (Thermo Fisher Scientific) and separated for analysis on an analytical reverse-phase Acclaim PepMap 100 C18, nanoViper, 3 μm, 75 μm × 25 cm column (Thermo Fisher Scientific) at a flow rate of 0.3 μL/min. The measurement method was chosen according to the sample and labeling type.

(1) Enriched acetyl-peptides and the corresponding complementary full proteomes were measured as previously described for label-free quantification (LFQ) sample analysis.^86^ Briefly, peptides were eluted over a 170 min three-step linear gradient starting at 4% solvent B (solvent A: 0.1% FA in water; solvent B: 80% ACN/0.1% FA in water), via 30% B after 95 min, 55% B after 135 min reaching 99% B after 150 min followed by flushing of the column for 5 min at 99% B and subsequent equilibration to initial conditions. The MS1 spectra at a scan range of 350–1550 *m*/*z* were acquired in the orbitrap positive mode with an automatic gain control (AGC) target of 3 × 10^6^ ions at a resolution of 120,000 and a maximum injection time (maxIT) of 100 ms. An isolation window of 1.4 *m*/*z* was used to select the top 10 precursors for fragmentation via collision-induced dissociation (CID) at a normalized collision energy (NCE) of 28. The gained MS2 spectra were recorded at a resolution of 15,000 with a maxIT of 100 ms and AGC target set to 2 × 10^6^. Dynamic exclusion of selected ions was set to 20 s.
(2) Enriched phosphopeptides were eluted and measured analogously to Karkossa, Fürst, Großkopf, von Bergen, and Schubert.^87^ In short, phosphopeptides were eluted over a 170 min four-step linear gradient starting at 4% solvent B (solvent A: 0.1% FA in water; solvent B: 80% ACN/0.1% FA in water), via 18% B after 78 min, 30% B after 115 min, 55% B after 145 min reaching 99% B after 150 min followed by flushing of the column for 5 min at 99% B and subsequent equilibration to initial conditions. Settings for the acquisition of positive mode full MS scans were the following: scan range of 350–1550 *m*/*z*, AGC target of 3 × 10^6^ ions, resolution of 120,000, and a maxIT of 150 ms. The top 15 precursor ions were selected for fragmentation, applying a 0.7 *m*/*z* isolation window and an NCE of 34 for CID fragmentation. Fragment ion spectra were recorded at the following settings: resolution of 60,000, maxIT of 150 ms, AGC target of 2 × 10^5^, and a dynamic exclusion of 45 s.
(3) TMT-labeled TPP samples were analyzed as described before.^86^ In brief, peptides were eluted over a 180 min three-step linear gradient starting at 4% solvent B (solvent A: 0.1% FA in water; solvent B: 80% ACN/0.1% FA in water), via 30% B after 100 min, 55% B after 140 min reaching 99% B after 150 min followed by flushing of the column for 5 min at 99% B and subsequent equilibration to initial conditions. Settings for the acquisition of positive mode full MS scans were the following: scan range of 350–1550 *m*/*z*, AGC target of 3 × 10^6^ ions, resolution of 1,20,000, and a maxIT of 120 ms. The top 15 precursor ions were selected for fragmentation, applying a 0.7 *m*/*z* isolation window and an NCE of 34 for CID fragmentation. Fragment ion spectra were recorded at the following settings: resolution of 60,000, maxIT of 120 ms, AGC target of 1 × 10^5^, and a dynamic exclusion of 45 s.
(4) All murine tissue and human SVF samples were analyzed in data-independent acquisition (DIA) on the same instrumental setup. Peptides were eluted over a 75 min three-step linear gradient starting at 4% solvent B (solvent A: 0.1% FA in water; solvent B: 80% ACN/0.1% FA in water), via 30% B after 52 min, 55% B after 60 min reaching 99% B after 62 min followed by flushing of the column for 5 min at 99% B and subsequent equilibration to initial conditions. The MS1 spectra at a scan range of 350–1407 *m*/*z* were acquired in the Orbitrap positive mode with an automatic gain control (AGC) target of 1 × 10^6^ ions at a resolution of 45,000 and a maximum injection time (maxIT) of 20 ms. An isolation window of 25 *m*/*z* and a fixed first mass of 200 *m*/*z* were used via collision-induced dissociation (CID) at a normalized collision energy (NCE) of 28. Loop count was set to 44, MSX count to 1, and MS2 spectra were recorded at a resolution of 45,000 with a maxIT of 50 ms and AGC target set to 3 × 10^6^.

The collected mass spectrometry proteomic data have been deposited to the ProteomeXchange Consortium via the PRIDE^88^ partner repository with the data set identifiers: PXD059419 (SVF data), PXD059386 (TPP with MINCH), PXD059428 (MINCH-exposed SGBS proteome), PXD059416 (MINCH-exposed SGBS phosphoproteome), PXD059390 (MINCH-exposed SGBS acetylome), and PXD059421 for the tissue proteome, phosphoproteome, and acetylome data.

### Protein Database Searches and Data Analysis

Raw files from data-dependent acquisition were searched using Proteome Discoverer (2.5.0.400, Thermo Fisher Scientific) against the UniProtKB reference proteome of *Homo sapiens* (downloaded 24.01.2024, 20,433 entries), respectively. Carbamidomethylation of cysteine (C) residues was assigned as a fixed modification, whereas oxidation of methionine (M) and N-terminal acetylation were designated as variable modifications. For samples enriched with acetylated or phosphorylated peptides, acetylation of lysine (K) and phosphorylation of serine (S), threonine (T), and tyrosine (Y) were included as additional variable modifications. For the TMT-labeled TPP samples, a TMT-label at lysine (K) was additionally added as static modification, as well as a TMT-label at the *N*-terminus as additional variable modification. The false discovery rate (FDR) for peptide, protein, and site identifications was set to 0.01, applying a target-decoy strategy using a reversed decoy database. Up to four missed tryptic cleavages were permitted for enriched acetylated peptide samples to account for cleavage interference by acetylated lysines, while for other samples, this parameter was limited to two. Protein identification required at least two peptides, one of which had to be unique. Quantification was based on the intensities of unique and razor peptides. Sites identified with confidence below the high level reported by the search engine (Sequest HT) were omitted from the analysis. Proteins and sites present in at least 3/4 of replicates were included for further examination, with site abundances normalized to their corresponding protein levels.

Raw files from data-independent acquisition were searched using Spectronaut 18 (Biognosys, Switzerland) in a directDIA analysis using the UniProtKB reference proteome of *Homo sapiens* (downloaded 24.01.2024, 20,433 entries) or *Mus musculus* (downloaded 24.01.2024, 17,201 entries). Otherwise, default settings were used. For the human SVF data set, proteins quantified in at least 5 out of 7 replicates were considered reliably quantified and used for further analysis. For the tissue proteomes, proteins quantified in all three replicates were considered for further analysis of the full proteome. Quantification in two-thirds of replicates was set as a threshold for further consideration for acetylome and phosphoproteome analysis.

R v4.3.0 was used for statistical analysis according to the workflow described for the analysis of label-free samples in the package *proteomicsr* v1.0.0.^89^ Further packages used were: *ggplot2* (v3.5.1),^90^ *corrplot* (v0.92), *limma* (v3.56.2), *plyr* (v1.8.9),^91^ *reshape2* (v1.4.4),^92^ *DEP* (v1.22.0),^93^ *ggsci* (v3.0.0),^94^ *pheatmap* (v1.0.12), *circlize* (v0.4.15), and *ClassDiscovery* (v3.4.0). Normalized site intensities were log_2_ transformed and variance stabilized. Mean intensities were computed to calculate fold changes (FC) for the different treatment conditions compared to the respective controls. A two-sided Student’s *t*-test with FDR adjustment for multiple testing was used to calculate significant changes in abundance (*p* value ≤ 0.05). All original data can be found in the PRIDE submissions and the Supporting Information (XLSX).

### Biological Network Inference, Clustering, Enrichment, and Data Correlation

Soft clustering of the differentially abundant protein profiles was performed using the R package *Mfuzz* v2.62.0.^95^ The number of clusters was set to 10, the estimated fuzzifier was determined as 1.765, and protein abundance profiles with cluster membership values >0.2 were plotted.

A *weighted gene correlation network analysis* (WGCNA) of proteome, acetylome, and phosphoproteome in response to distinct differentiation conditions was performed using the corresponding R package *WGCNA* ^32,96^ on log_2_ transformed protein as well as corrected acetyl- and phosphosite intensities. The network was constructed based on the following parameters: a soft thresholding power of 11, minimum/maximum module size of 50/300, a deepsplit value of 2, and a merge cut height of 0.5, rendering 12 modules for data analysis.

A protein set enrichment analysis of Mfuzz cluster and WGCNA module members on GOBP and KEGG terms, GOCC compartments, and UniProt Keywords was performed using functional protein association networks built by STRING v12.0.^97^

The tool *SubcellulaRVis* ^37^ was used to enrich for subcellular compartments using Gene Ontology Cellular Components (GOCC). A gene set enrichment analysis of proteome, acetylome, and phosphoproteome was performed using the MSigDataBase (MSigDB; gsea-msigdb.org/gsea/msigdb/collections.jsp) with the respective species (*Homo sapiens* or *Mus musculus*) and Gene Ontology Biological Processes (GOBP) and Kyoto Encyclopedia of Genes and Genomes (KEGG) curated gene sets. The original data of STRING and MSigDB enrichment analyses can be found in the Supporting Information (XLSX).

### Analysis of Protein Interaction Partners by Thermal Shifts

Melting points were determined from the generated melting curves as described previously.^38^ In brief, the relative abundances of the TMT reporter ion intensities were calculated in comparison to the lowest incubation temperature. Hence, the lowest incubation temperature obtained a relative abundance of 1. The data was normalized and processed using the TPP-TR script in R (v4.3.0),^38,98^ applying the following denaturation equation (*T*, temperature; a/b/plateau are constants):

$$f\left(T\right)=\frac{1-plateau}{1+e^{-\left(\frac{a}{T}-b\right)}}+plateau$$

The melting point of a protein (*T*_m_) is defined as the temperature where the relative abundance of the protein is half its initial value; *f*(*T*_m_) = 0.5. The determined protein melting points from vehicle- and MINCH-treated lysates were tested for significance with *p* < 0.05 by applying a two-sided Student’s *t-*test. In addition, interaction candidates were further curated using the following criteria: (1) a curve fit with *R* ^2^ > 0.9 for all replicates and (2) a melting point shift indicating the same direction for all pairwise solvent vs treatment comparisons. Melting curves of the remaining hits were manually inspected. Candidate melting curves were visualized in GraphPad Prism (v9.4.1). A summary of the melting temperature (*T*_m_) of all quantified proteins can be found in the Supporting Information (XLSX).

## Supplementary Material





## Data Availability

All proteomics data can be accessed via PRIDE with the project identifiers PXD059419 (SVF data), PXD059386 (TPP with MINCH), PXD059428 (MINCH-exposed SGBS proteome), PXD059416 (MINCH-exposed SGBS phosphoproteome), PXD059390 (MINCH-exposed SGBS acetylome), and PXD059421 for the tissue proteome, phosphoproteome, and acetylome data. All original code is available upon request.

## Notes

Resources table; distribution of animal numbers across the different experimental groups; complete cluster enrichment; Pearson correlation analysis between the proteomes of SGBS and SVF adipocytes; curated sets of TPP candidate curves; KinSwing kinase prediction longlist; lipid accumulation in 3T3-L1 and primary murine adipose eWAT/iWAT SVF cells after exposure to DINCH and MINCH; enrichment analysis on differentially abundant proteins, acetyl- and phospho-proteins between HFD- vs SD (chow)-fed C57BL/6N mice and those exposed to DINCH-containing HFDs; correlation analysis of visceral (VIS) and subcutaneous (SC) proteome tissue data (PDF)
Replicate data on quantified proteins, log_2_(FCs) and *p* values for all reliably quantified proteins of (1) human subcutaneous adipose tissue SVF data, (2) proteome, (3) acetylome, and (4) phosphoproteome of differentiated SGBS cell under different exposure conditions, (5) tissue proteome, (6) acetylome, and (7) phosphoproteome of SC and VIS adipose tissue of C57BL/6N mice fed standard high fat or DINCH contaminated high-fat diet; sample replicate data, replicate TPP melting temperatures (Tm) and *p* values for all reliably quantified proteins, thermal proteome profiling of SGBS cells under solvent/MINCH incubation; *Mfuzz* cluster memberships of SVF proteome data; enrichment analyses data of human subcutaneous adipose tissue SVF global proteome using the STRING database, cluster enrichments shown for *Mfuzz* clusters and the databases GOBP, GOCC, KEGG and UniProt Keywords; enrichment analyses data of differentiated SGBS cell under different exposure conditions global proteome, acetylome and phosphoproteome data, median log2­(FCs) and *p* values for all pathways and the two databases GOBP and KEGG; enrichment analyses data of tissue proteome, acetylome, and phosphoproteome of SC and VIS adipose tissue of C57BL/6N mice fed standard high-fat or DINCH contaminated high-fat diet, median log2­(FCs) and *p* values for all pathways and the two databases GOBP and KEGG (XLSX)
