# Supplementary material for: Advanced Proteomics Approaches Hold Potential for the Risk Assessment of Metabolism-Disrupting Chemicals as Omics-Based NAM: A Case Study Using the Phthalate Substitute DINCH
Source: Environ Sci Technol. 2025 Jul 30;59(31):16193–216. doi: 10.1021/acs.est.5c01206 (PMC12355959; doi:10.1021/acs.est.5c01206)
Supplement: Supplementary file 1 [file es5c01206_si_001.pdf]

Supporting Information (SI) | Figures and Tables

Article Title

Advanced proteomics approaches hold potential for the risk assessment of metabolism disrupting chemicals as omics-based NAM – a case study using the phthalate substitute DINCH

Authors

Alix Sarah Aldehoff [1], Isabel Karkossa [1], Helen Broghammer [2], Sontje Krupka [2,3], Juliane Weiner [2,3], Cornelius Goerdeler [1], Rima Nuwayhid [4], Stefan Langer [4], Martin Wabitsch [5,6], Ulrike Rolle-Kampczyk [1], Nora Klöting [2], Matthias Blüher [2,3,6], John T. Heiker [2,7], Martin von Bergen [1,6,7,8,9] and Kristin Schubert [1,9*]

Affiliations and Contact Information

[1] Department of Molecular Systems Biology, Helmholtz-Centre for Environmental Research GmbH (UFZ), 04318 Leipzig, Germany

[2] Helmholtz Institute for Metabolic, Obesity and Vascular Research HI-MAG, Helmholtz-Centre Munich at the University of Leipzig and University Hospital, 04103 Leipzig, Germany

[3] Department of Medicine, Endocrinology and Nephrology, University of Leipzig, 04103 Leipzig, Germany

[4] Department of Orthopaedic, Trauma and Plastic Surgery, Division of Plastic, Aesthetic and Special Hand Surgery, University Hospital Leipzig, 04103 Leipzig, Germany

[5] Division of Paediatric Endocrinology and Diabetes, University Hospital for Children and Adolescents Ulm, 89075 Ulm, Germany

[6] German Centre for Child and Adolescent Health (DZKJ), 04103/04318 Leipzig, Germany

[7] Institute of Biochemistry, Faculty of Biosciences, Pharmacy and Psychology, University of Leipzig, 04103 Leipzig, Germany

[8] German Centre for Integrative Biodiversity Research (iDiv) Halle-Jena-Leipzig, 04103 Leipzig, Germany

[9] Authors contributed equally

[*] Correspondence: kristin.schubert@ufz.de

Contents

Number of Manuscript Pages: **64**

Number of Main Figures: **7**

Number of Supporting Information Figures: **11**

Number of Main Tables: **0**

Number of Supporting Information Tables: **2** (**PDF**) and **12** (**XLSX**)

Main Supplemental File

Table S1 | Resources table.

Reagent or Resource	Source	Identifier
**Experimental models**		
Mouse C57BL/6NTac	Taconic Biosciences	RRID:IMSR_TAC:B6
**Biological samples**		
Human subcutaneous SVF cells	University Clinic of Leipzig	N/A
**Cell line**		
SGBS	Martin Wabitsch, Wabitsch *et al.* 2001	RRID:CVCL_GS28
**Chemical and reagents**		
DMEM/F12	Gibco ™	Cat# 11554546
Biotin	Sigma-Aldrich	Cat# B4639
Panthothenate	Sigma-Aldrich	Cat# PHR1232
Penicillin/streptomycin	Sigma-Aldrich	Cat# P4333
Fetal Bovine Serum	Gibco ™	Cat# A3160802
Cortisol	Sigma-Aldrich	Cat# H0888
apo-transferrin	Sigma-Aldrich	Cat# T2036
Triiodothyronine	Sigma-Aldrich	Cat# T6397
Insulin	Sigma-Aldrich	Cat# I2643
Rosiglitazone	Sigma-Aldrich	Cat# R2408
Dexamethasone	Sigma-Aldrich	Cat# D1756
3-isobutyl-1-methylxanthine	Sigma-Aldrich	Cat# I5879
Collagenase, Type II	Gibco ™	Cat# 10738473
ZellShield®	Minerva biolabs	Cat# 13-0050
GW9662	Cayman Chemical Company	Cat# 70785
MINCH, mono-isononyl-cyclohexane-1,2-dicarboxylate	Toronto Research Chemicals	Cat# C987305
DINCH, diisononyl-cyclohexane-1,2-dicarboxylate	abcr	Cat# AB440048
AdipoRed™ Assay Reagent	Lonza	Cat# PT-7009
Saponin	Sigma-Aldrich	Cat# 47036
PBS	biowest	Cat# L0625-500
Paraformaldehyde	Sigma-Aldrich	Cat# 158127
HEPES	Roth	Cat# 6763.3
Urea	Merck	Cat# 108487
Sodium orthovanadate	Sigma-Aldrich	Cat# 567540
Sodium pyrophosphate dibasic	Sigma-Aldrich	Cat# P8135
ß-glycerophosphate	Alfa Aesar	Cat# L03425
Dithiothreitol	GE Healthcare	Cat# GE17-1318-02
2-Iodoacetamid	Merck	Cat# 804744
Sequencing Grade Modified Trypsin	Promega	Cat# V5111
TCEP	Sigma-Aldrich	Cat# C4706
Formic acid	Fluka Honeywell	Cat# 15658430
Hydroxylamine	Thermo Fisher Scientific	Cat# 90115
Trifluoroacetic acid	Merck	Cat# 302031
Acetonitrile	Roth	Cat# T195.2
DMSO	Sigma-Aldrich	Cat# D2650
MS grade water	Supelco	Cat# 1.15333
Ammonium formiat	Sigma-Aldrich	Cat# 156264
Ethanol	Merck	Cat# 111727
Methanol	Promochem	Cat# SO9356
cOmplete™ protease inhibitor cocktail	Roche	Cat# COEDTAF-RO
Sodium chloride	Sigma-Aldrich	Cat# S9888

**Commercial kits**		
Pierce™ 660 nm Protein-Assay-Reagent	Thermo Fisher Scientific	Cat# 22660
DC™ Protein assay	BioRad	Cat# 5000112
PTMScan® Acetyl-Lysine Motif [Ac-K] Kit	Cell Signaling Technology	Cat# 13416
High-Select™ TiO2 Phosphopeptide Enrichment Kit	Thermo Fisher Scientific	Cat# A32993
High-Select™ Fe-NTA Phosphopeptide Enrichment Kit	Thermo Fisher Scientific	Cat# A32992
TMT10plex™	Thermo Fisher Scientific	Cat# 90111
**Software**		
Proteome Discoverer v2.5.0.400	Thermo Fisher Scientific	N/A
Spectronaut®18	Biognosys	N/A
R v4.3.0	The R Project for Statistical Computing	https://www.r-project.org/
Inkscape v0.92.4	INKSCAPE	https://inkscape.org/
GraphPad Prism v9.4.1	GraphPad Software	https://www.graphpad.com/
Microsoft Office	Microsoft	N/A
PROVIEW version x64	VWR	N/A
Gen5 version 2.06	Bio-Tek	N/A
**Data**		
Human reference proteome, reviewed	UniProt, downloaded 24.01.2024	https://www.uniprot.org/
Mouse reference proteome, reviewed	UniProt, downloaded 24.01.2024	https://www.uniprot.org/
Protein mass spectrometry data	This paper (PRIDE), Identifier PXD059386, 10.6019/PXD059386 (TPP data); PXD059428, 10.6019/PXD059428 (SGBS MINCH proteome); PXD059390, 10.6019/PXD059390 (SGBS MINCH acetylome); PXD059416, 10.6019/PXD059416 (SGBS MINCH phosphoproteome); PXD059419 (SVF data); PXD059421 (tissue proteome, phosphoproteome and acetylome data)	https://www.ebi.ac.uk/pride/
**Hardware and others**		
Syringe strainer, 300 µm	puriSelect®	Cat# 43-71300-51
MACS® SmartStrainer, 30 µm	Miltenyi Biotec	Cat# 130-110-915
Thermocycler, G-STORM	Alpha Metrix Biotech	Cat# GS002M
Chow diet, V1534	Ssniff	N/A
High fat diet, E15772-34	Ssniff	N/A
Oasis HLB 1cc, 30 mg, Vac Cartridges	Waters	Cat# WAT094225
SpeedBead Magnetix Carboxylate Beads	Cytiva	Cat# GE65152105050250
VisiScope® IT415 PH, Inverted Microscope	VWR	Cat# 630-3098
Thermomixer	Eppendorf	Cat# 5355 000.011
Platereader Synergy HT	Bio-Tek	Cat# 195232
SpeedVac Concentrator Plus	Eppendorf	Cat# EP5305000100-1EA
Lyophylle Alpha 2-4 LSC	Christ	Cat# 102142
UltiMate™ 3000 RS HPLC	Thermo Fisher Scientific	Cat# IQLAAAGABHFAPBMBEZ
Thermo Scientific™ Q Exactive™ HF Hybrid Quadrupole-Orbitrap™ Mass Spectrometer	Thermo Fisher Scientific	Cat# IQLAAEGAAPFALGMBFZ
TriVersa NanoMate	Advion	Cat# TR263
Acclaim PepMap 100 C18 NanoViper column 3 µm, 75 µm x 2 cm	Thermo Fisher Scientific	Cat# 164946
Acclaim PepMap 100 C18 NanoViper column 3 µm, 75 µm x 25 cm	Thermo Fisher Scientific	Cat# 164569

Table S2 | Distribution of animal numbers across the different experimental groups. Lysate pooling for subsequent proteome, phosphoproteome and acetylome analysis in technical triplicates of visceral/epididymal and subcutaneous/inguinal adipose tissues of C57BL/6N mice after dietary DINCH exposure.

	**Treatment**	
	**Female**	**Male**
**Standard Diet**	8	8
**High Fat Diet**	7	8
**High Fat Diet with low DINCH**	7	7
**High Fat diet with high DINCH**	8	8
**Total per sex**	30	31
**Total**	**61**	

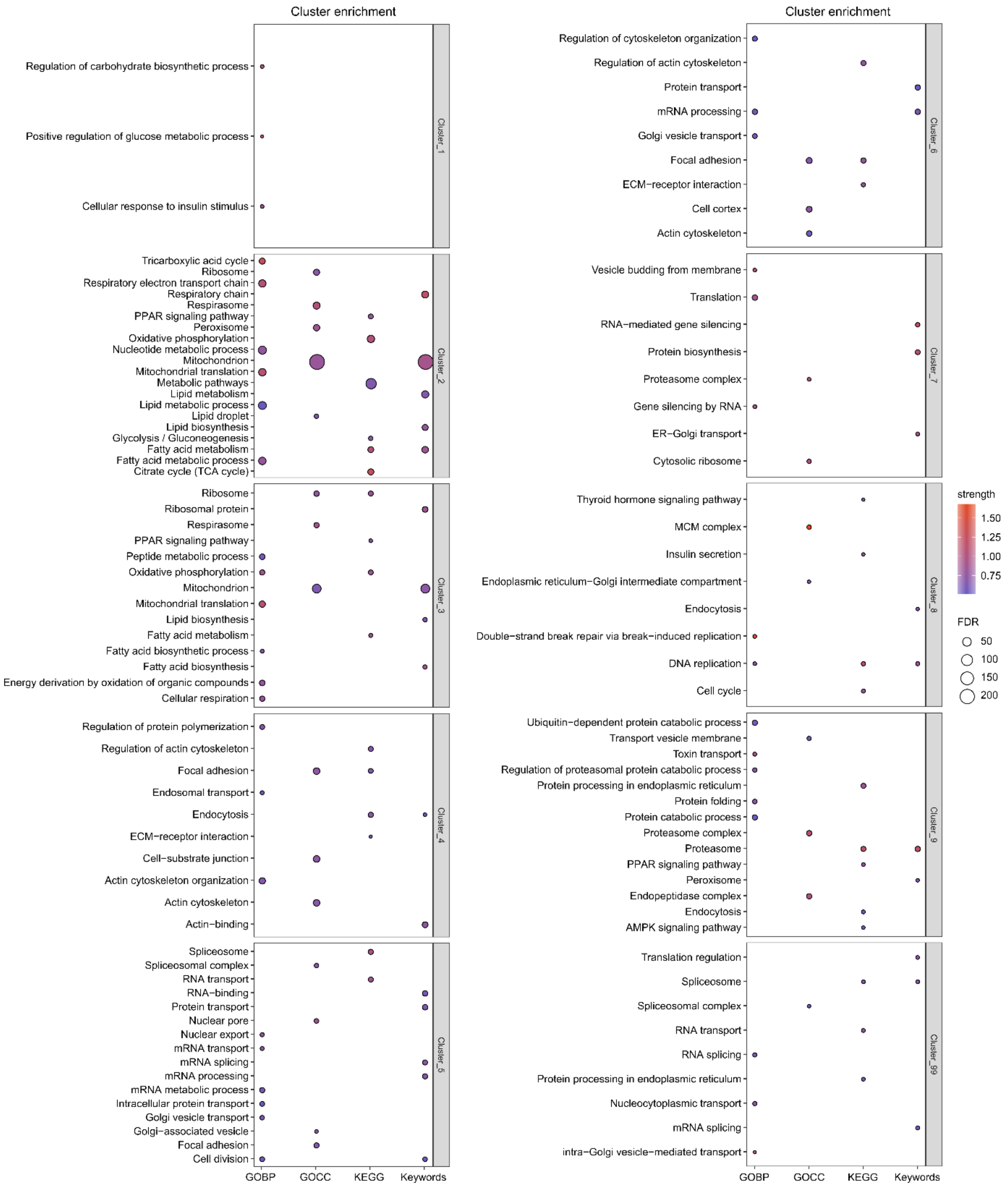

Figure S1 | Complete cluster enrichment. STRING enrichment of proteins assigned to all 10 clusters using GOBP, GOCC, KEGG terms and UniProt Keywords. Significantly enriched terms and keywords are displayed as strength of enrichment and -$\log_{10}$(FDR).

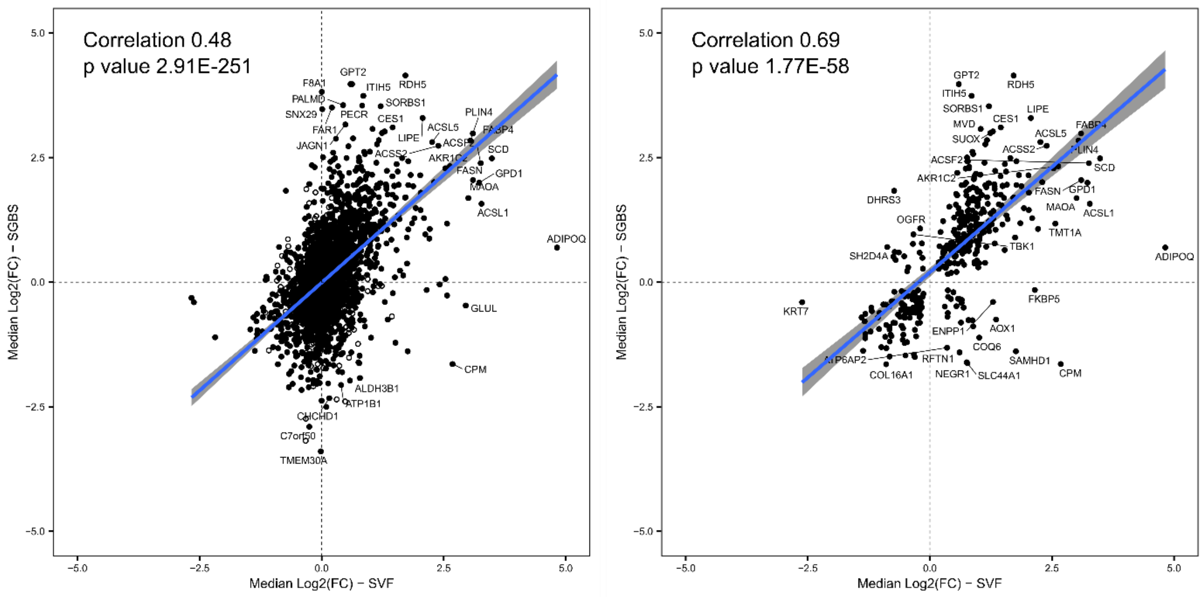

Figure S2 | Pearson correlation analysis between the proteomes of SGBS and SVF adipocytes exposed to 10 µM MINCH during adipogenesis. In the left plot, all proteins were included in the analysis. Those with a significant change in abundance (adj. *p* value < 0.05) in response to the chemical treatment are displayed as filled dots. In the plot at the right, only proteins with a significant change in abundance (adj. *p* value < 0.05) in both cell models in response to 10 µM MINCH during adipogenesis are displayed and included in the correlation analysis.

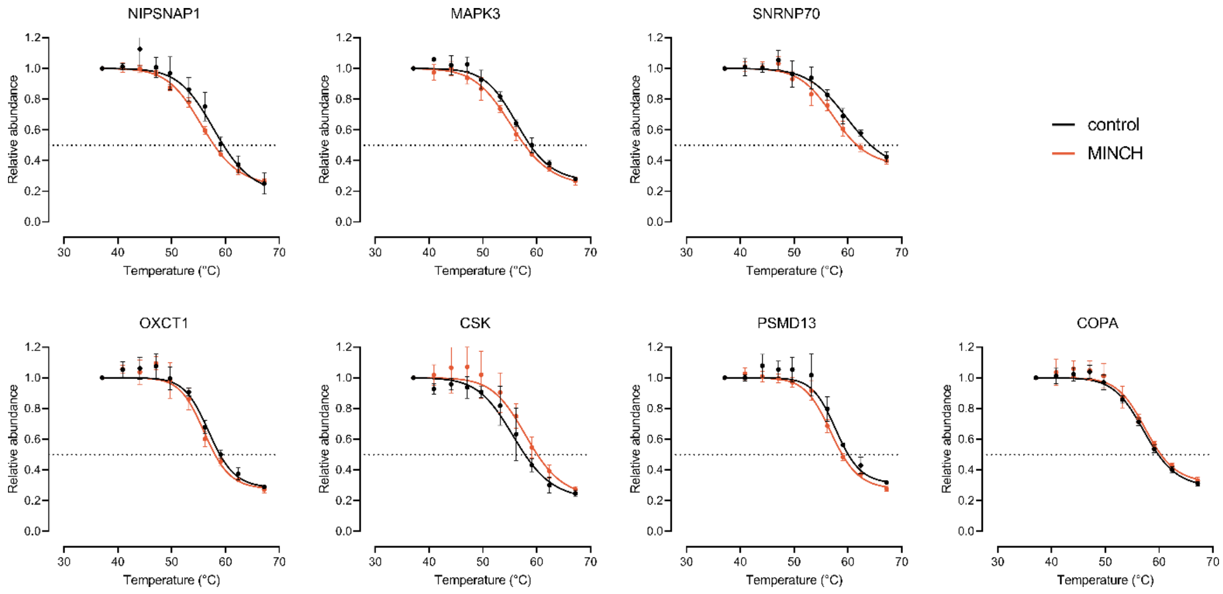

Figure S3 | Curated set of early TPP candidate curves.

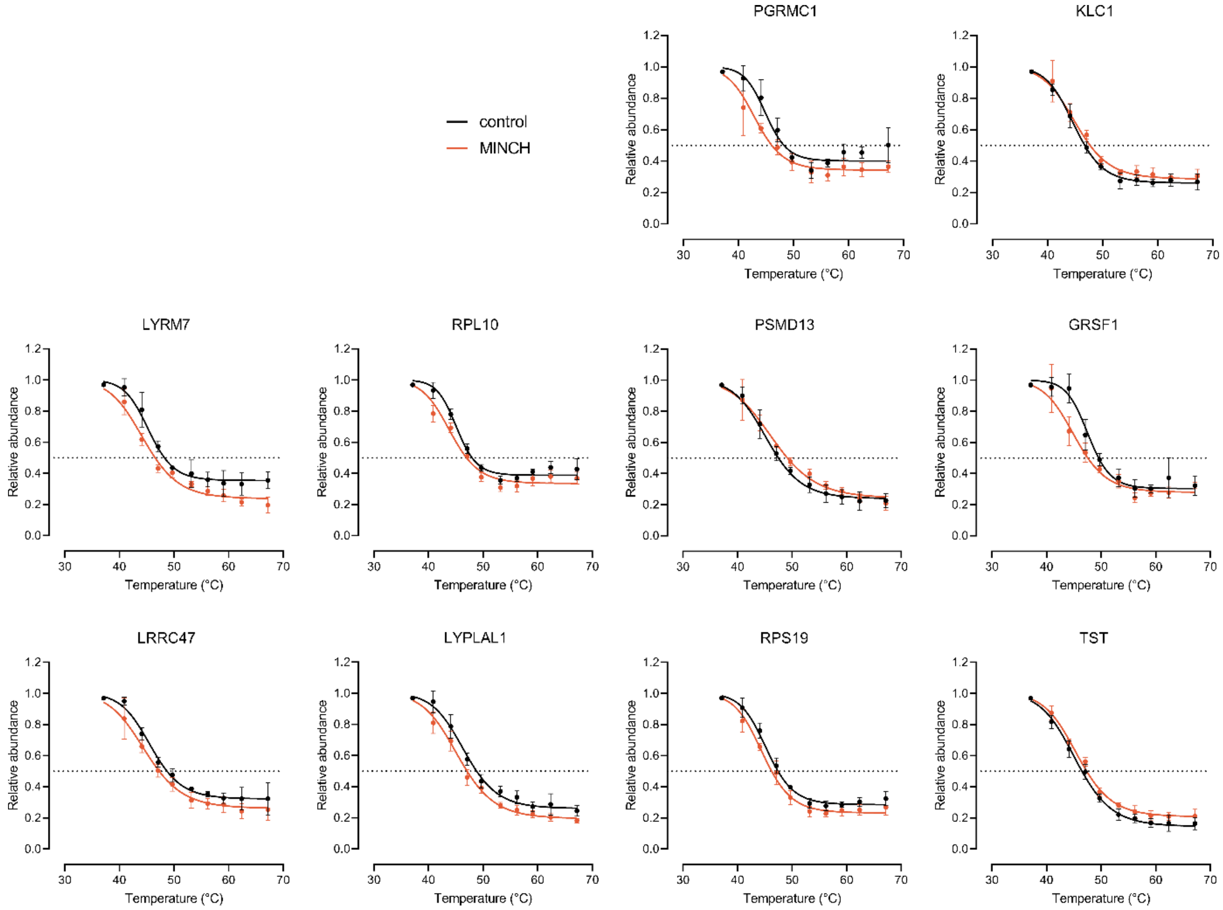

Figure S4 | Curated set of terminal TPP candidate curves.

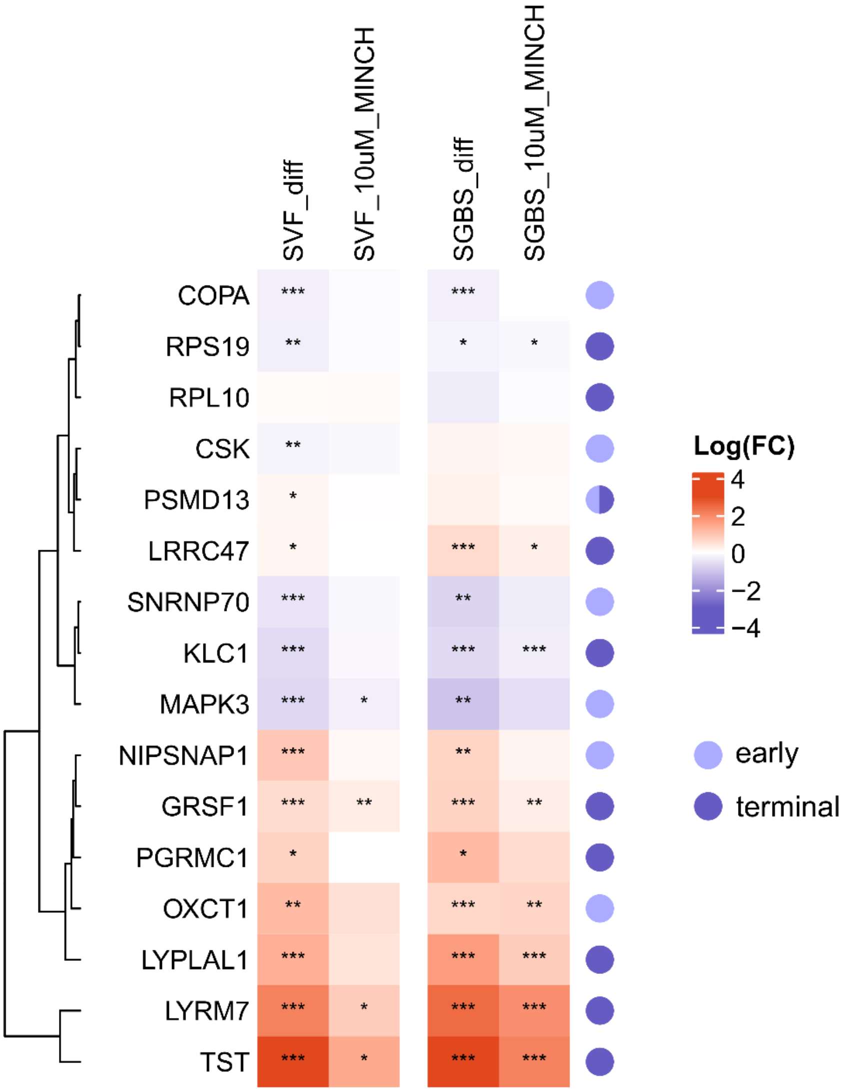

Figure S5 | Curated list of TPP candidates identified in lysates of SGBS adipocytes incubated with 10 µM MINCH. The respective changes in abundance at global proteome level report the comparison between rosiglitazone (diff) and MINCH (10 µM) exposed SVF and SGBS adipocytes during adipogenesis to the solvent control. TPP candidates identified in SGBS cell lysates from early (d4) and terminal (d12) differentiation are indicated on the right.

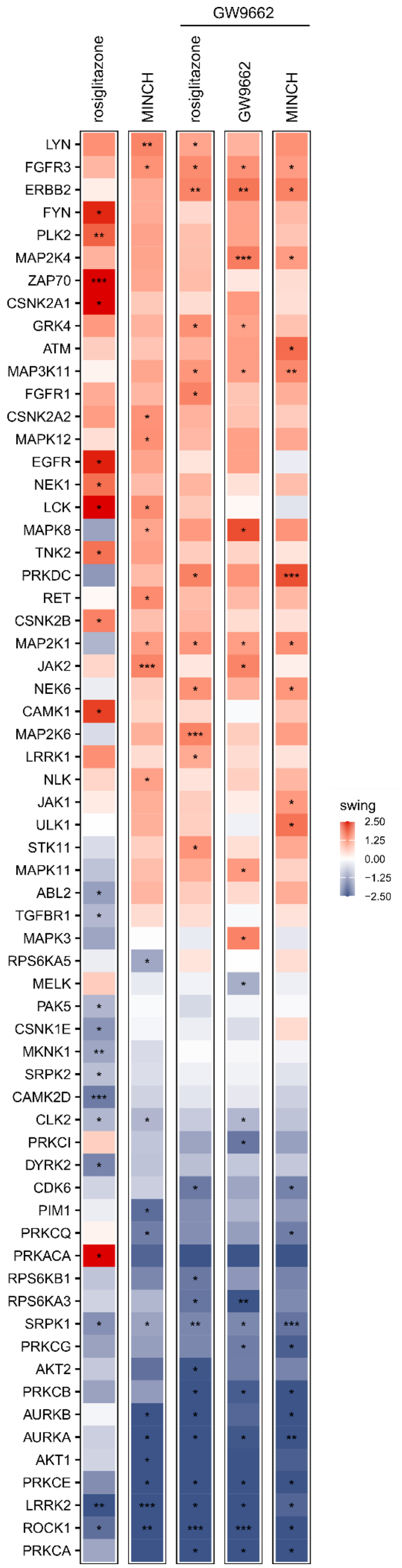

Figure S6 | KinSwing kinase prediction longlist.

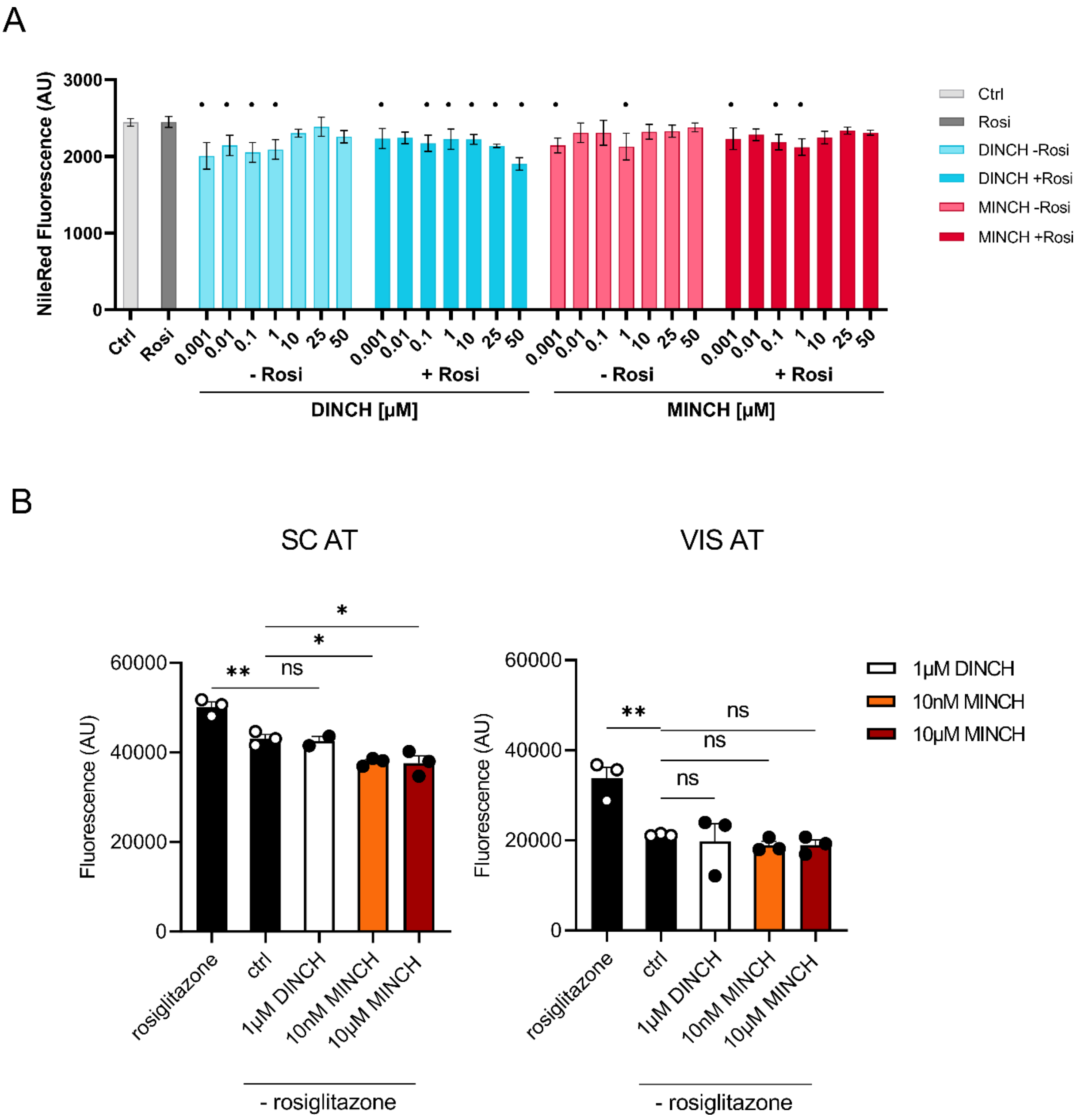

Figure S7 | Lipid accumulation in 3T3-L1 and primary murine adipose eWAT/iWAT SVF cells after exposure to DINCH and MINCH. A, Lipid accumulation in 3T3-L1 cells following DINCH/MINCH-initiated adipocyte differentiation. Significance calculated in comparison to the control (ctrl, solvent) and indicated as p value < 0.05, •. **B,** Lipid quantification in primary murine SVF cells following DINCH/MINCH exposure during differentiation. Cells derived from the subcutaneous (inguinal) and visceral (epididymal) adipose tissue of male C57BL/6N mice.

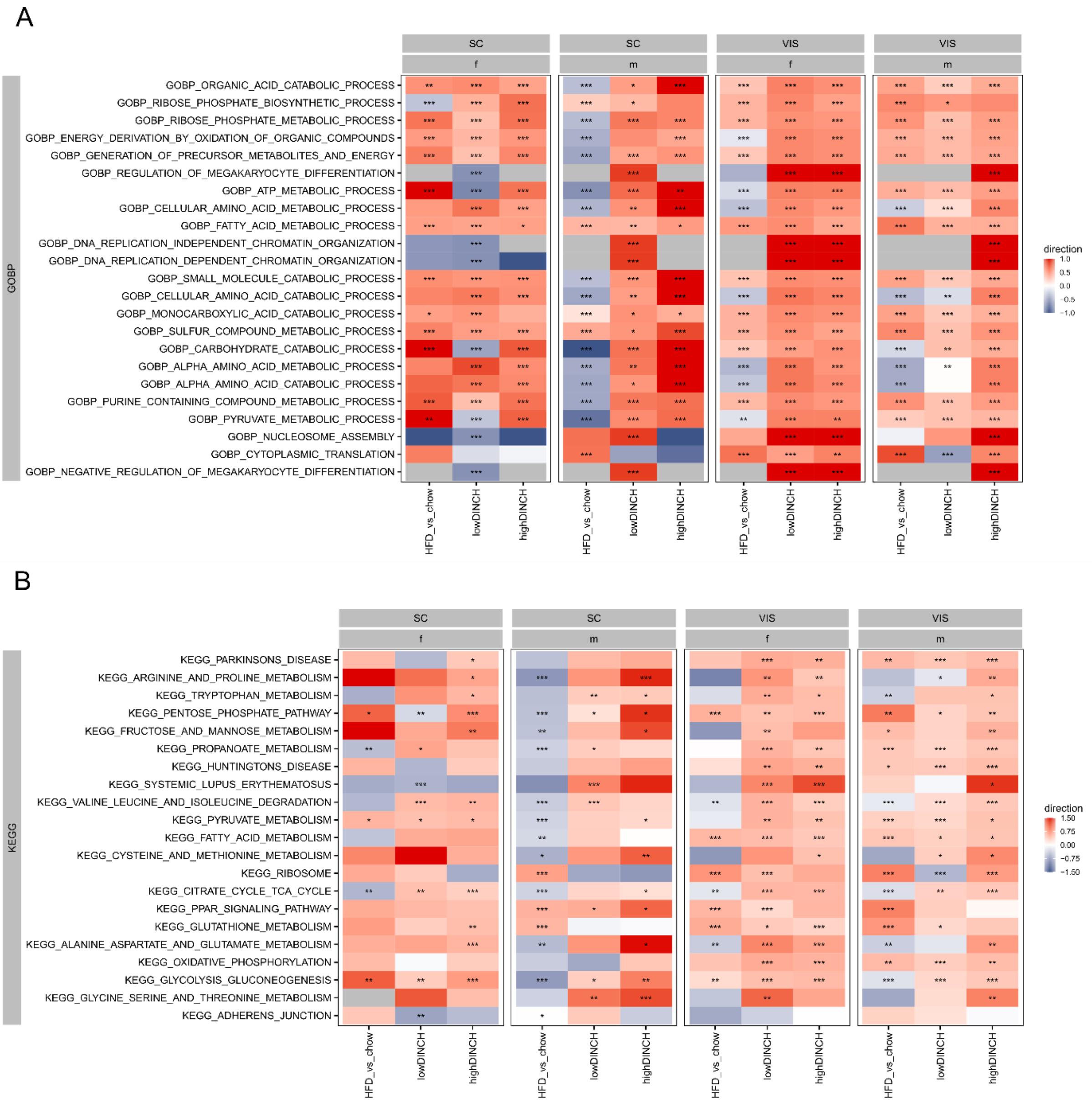

Figure S8 | Enrichment analysis on differentially abundant proteins between HFD- vs SD (chow)-fed C57BL/6N mice and those exposed to DINCH containing HFDs.

A

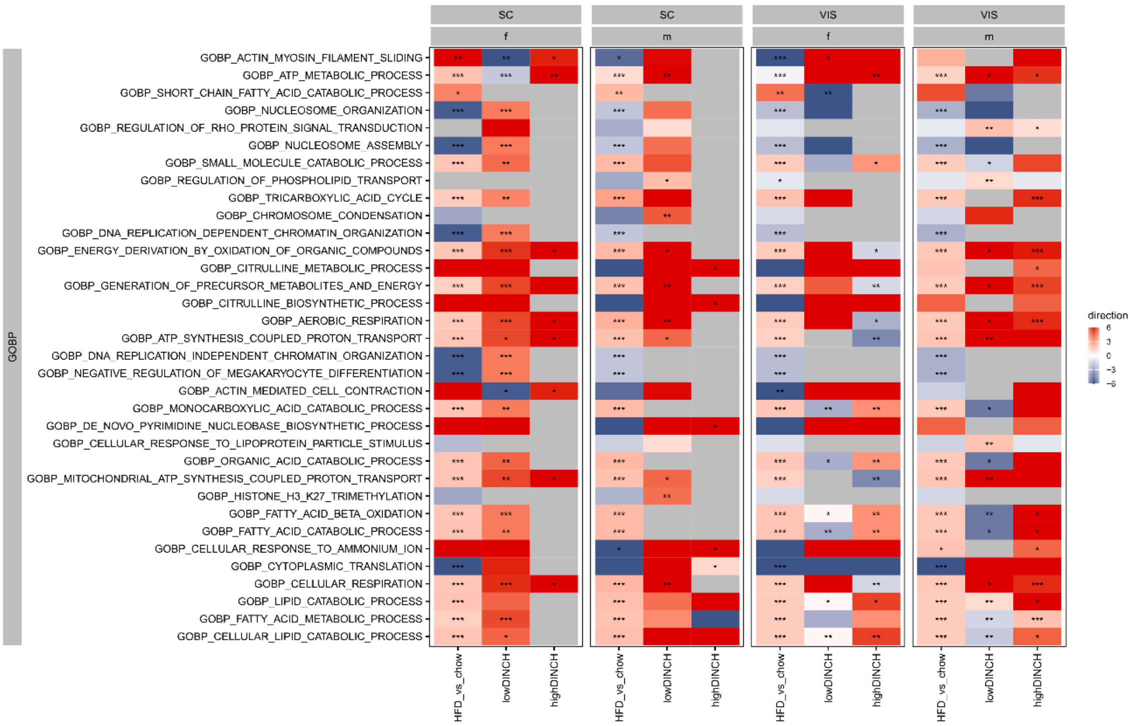

B

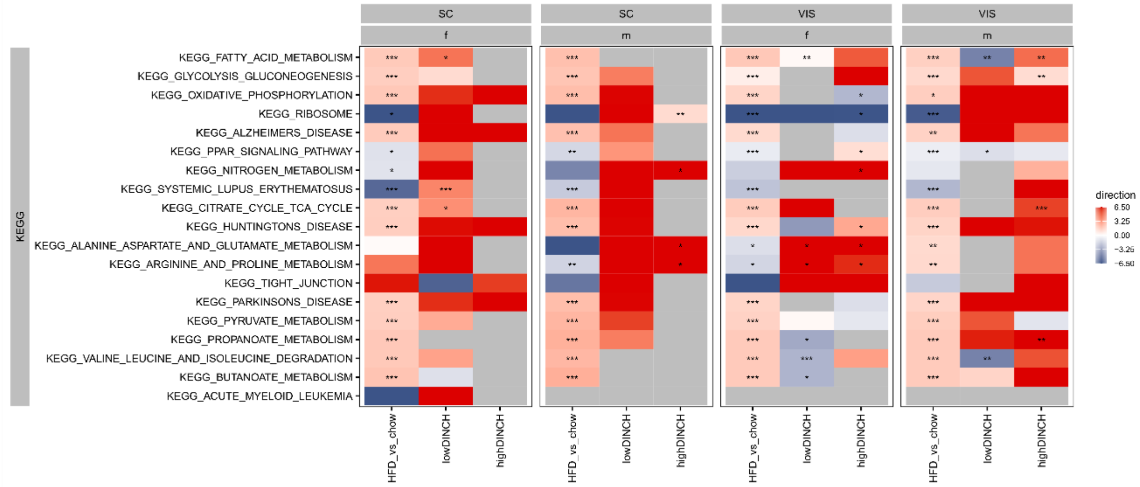

Figure S9 | Enrichment analysis on differentially abundant acetyl-proteins between HFD- vs SD (chow)-fed C57BL/6N mice and those exposed to DINCH containing HFDs.

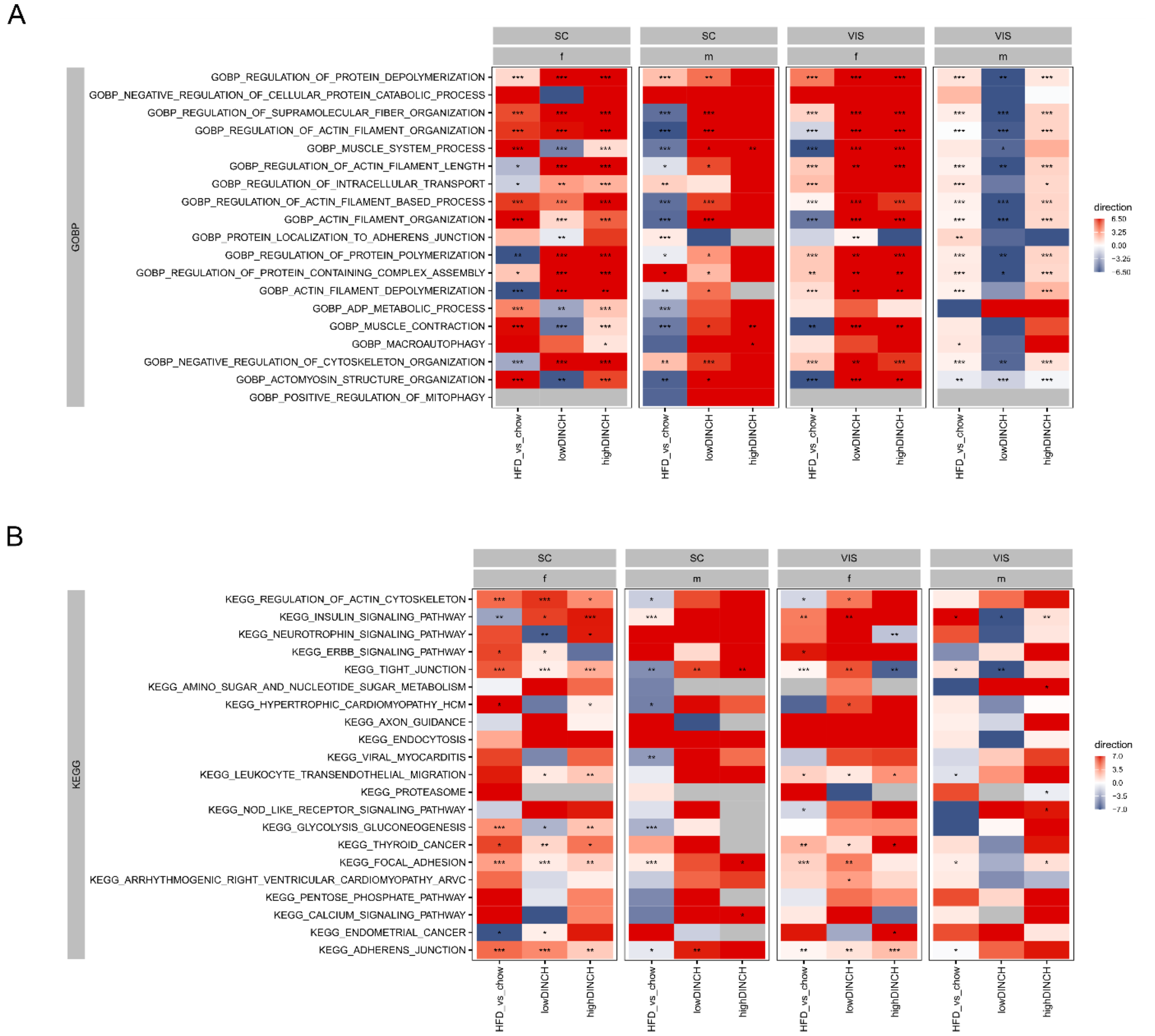

Figure S10 | Enrichment analysis on differentially abundant phosphoproteins between HFD- vs SD (chow)-fed C57BL/6N mice and those exposed to DINCH containing HFDs.

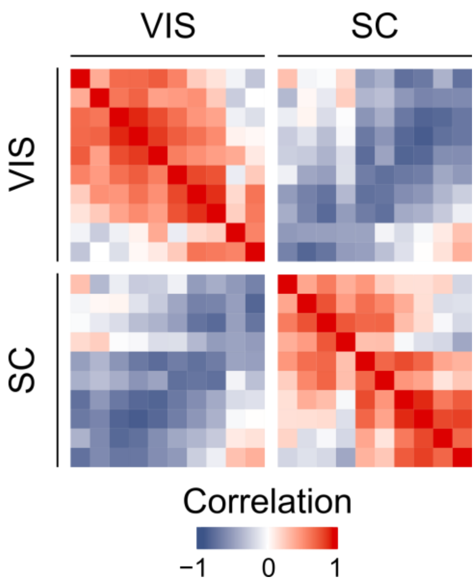

Figure S11 | Correlation analysis of visceral (VIS) and subcutaneous (SC) proteome tissue data. Integrated correlation of proteome, acetylome and phosphoproteome data including VIS and SC data from male and female mice on all four experimental diets SD, HFD, LD and HD.
